# Assisted death in eating disorders: a systematic review of cases and clinical rationales

**DOI:** 10.3389/fpsyt.2024.1431771

**Published:** 2024-07-31

**Authors:** Chelsea Roff, Catherine Cook-Cottone

**Affiliations:** ^1^ Eat Breathe Thrive, London, United Kingdom; ^2^ Department of Counseling, School, and Educational Psychology, University at Buffalo, Buffalo, NY, United States

**Keywords:** assisted dying, eating disorders, anorexia nervosa, medical assistance in dying, euthanasia, assisted suicide, severe and enduring eating disorders, severe and enduring anorexia

## Abstract

**Background:**

Assisted dying for reasons solely related to an eating disorder (ED) has occurred in multiple countries, including those which restrict the practice to individuals with a terminal condition. The aims of this systematic review were to (1) identify all known cases of assisted deaths among patients with EDs and (2) describe the clinical rationales used to grant patients’ requests for assisted death.

**Methods:**

We conducted a systematic search of peer-reviewed studies and publicly available government reports to identify cases of assisted death in patients with EDs. In reports that included qualitative data about the case, clinical rationales were extracted and grouped into domains by qualitative content analysis.

**Results:**

We identified 10 peer-reviewed articles and 20 government reports describing at least 60 patients with EDs who underwent assisted dying between 2012 and 2024. Clinical rationales were categorized into three domains: irremediability, terminality, and voluntary request. Reports emphasized that patients with EDs who underwent assisted death had terminal, incurable, and/or untreatable conditions and had adequate decision-making capacity to make a life-ending decision. Most government reports did not include descriptive-enough data to verify psychiatric conditions.

**Conclusion:**

The results of our systematic review underscore considerable gaps in the reporting of assisted death in patients with psychiatric conditions, posing substantial concerns about oversight and public safety. In many cases, the clinical rationales that were used to affirm patients with EDs were eligible for assisted death lack validity and do not cohere with empirical understanding.

## 1 Introduction

Over the last two decades, a growing number of states, provinces, and countries have legalized medical assistance in dying (1). Some jurisdictions now allow assisted dying for reasons solely related to a psychiatric disorder, which has catalyzed a complex debate about the ethical, legal, and medical basis for its use in patients with EDs (2–5). There is little reliable empirical data on how many patients with EDs have undergone assisted dying globally, but cases have been documented in the United States (2), the Netherlands (6–10), and Belgium (11–13).

The first reported physician assisted deaths of patients with anorexia nervosa in the United States came to light in 2022. In a case study published by Gaudiani et al. (2), the lead author and consulting physician prescribed lethal medications to two patients characterized as having *severe and enduring anorexia*, which the physician deemed *terminal* and *irremediable*. The authors proposed criteria for a new subcategory of anorexia, which they dubbed “*terminal anorexia*” (2 p. 2). They further stated this subgroup of patients “should be afforded access to medical aid in dying in locations where such assistance has been legalized—just like other patients with terminal conditions” (2 p. 12).

Currently, assisted dying is legal in 33 jurisdictions internationally. Reports of assisted death in patients with EDs are sparse in the peer reviewed literature, potentially related to the absence of legitimate legal pathways in most jurisdictions, substantial ethical issues for medical and mental health professionals, and limited data available to researchers in public reports (14). The criteria used to determine eligibility for assisted dying (e.g., terminal prognosis, irremediable condition) vary between jurisdictions, and research on their application to EDs is underdeveloped. Further, variation in eligibility criteria across jurisdictions makes it difficult for practitioners, academics, and governments to critically evaluate the legal and ethical basis of reported cases.

This systematic review aims to comprehensively identify all published cases of assisted death in patients with EDs and the clinical rationales that were used to justify its use in these patients. In order to facilitate understanding, we begin with a summary of the definitions and terms associated with assisted dying and the legal frameworks used to regulate assisted dying practices in jurisdictions around the world. The discussion section includes a critical examination of each rationale identified in the review and underscores gaps in the current literature to guide future research, clinical practice, and policymaking.

### 1.1 Assisted dying: definitions and terminology

*Assisted dying* refers to the practice of healthcare professionals prescribing or administering lethal drugs to end a patient’s life at their voluntary request, subject to eligibility criteria and safeguarding measures (15). Assisted dying is known by many names across different countries, and terminology is both evolving and a subject of debate (15). There is little agreement on definitions, and terms are often selected or created for the purpose of shaping public discourse (16). The terms *euthanasia*, *physician-assisted dying* and *physician-assisted death* are most common in Europe, and in North America, the term *medical aid in dying* (MAiD) is commonly used in public discourse (15). In the United States, *physician assisted suicide* is used in legislation, although recently there has been a shift toward use of the terms *MAiD* and *death with dignity* (15). In Australia, the term *voluntary assisted dying* is used most frequently. In some countries (e.g. Switzerland), physicians do not administer lethal medications, so the phrase physician-assisted is less frequently used (15).

The terms *assisted dying* and *assisted death* are often used to broadly encompass both assisted suicide and euthanasia (15). *Assisted suicide* involves patients self-ingesting lethal medications provided to them, while *euthanasia* entails a healthcare provider directly administering lethal medications, typically by injection (**Table 1**; 15). In this paper, we use assisted dying and assisted death to refer collectively to both methods of providing medical aid to end a person’s life upon their voluntary request. It is important to note that the involvement of physicians can vary; for example, in Switzerland, physicians are not directly involved in these practices (18). We employ jurisdiction-specific terminology when discussing particular use cases throughout this paper.

**Table 1:** Definitions and terms for assisted dying.

Commonly used terms	Definition
Assisted Dying or Death	The act of prescribing or administering lethal drugs to end a person’s life at their voluntary request.
Euthanasia	Directly *administering* life-ending drugs at the voluntary request of a patient, with the intention of ending life.
Assisted Suicide	*Prescribing* lethal drugs for patients to self-administer, with the intention of helping them to end their own life.

Informed by Hobbs and Gajjar (17) and Richardson (1).

### 1.2 Assisted dying: international legal status, eligibility criteria, and safeguards

**Figure 1:**
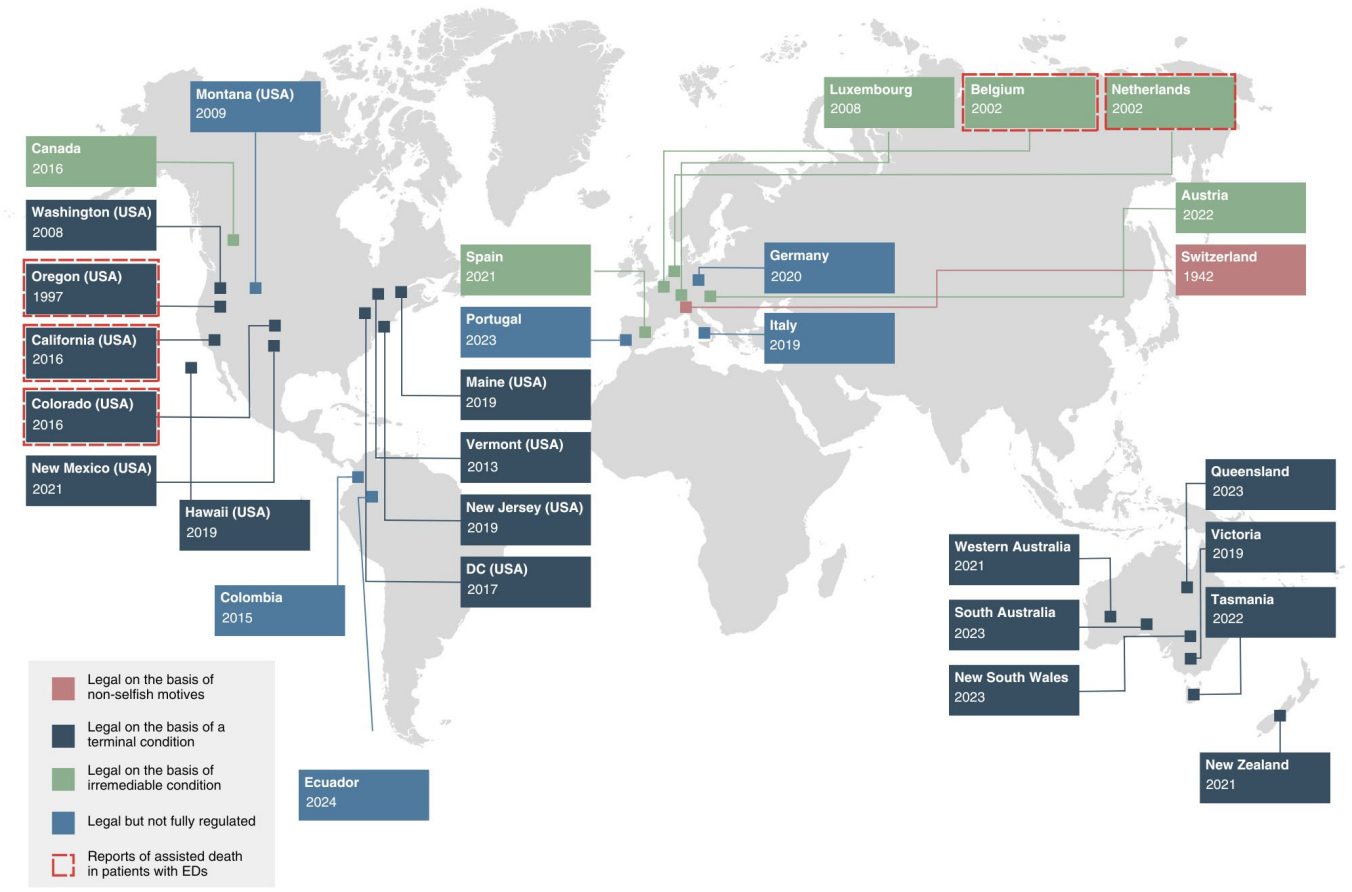
Legal basis of assisted dying around the world and reports of ED cases. Elements of this figure informed by Mroz et al. (15) and House of Commons Health and Social Care Committee (19).

The legalization of assisted dying has expanded considerably over the past two decades. Assisted dying is now legal in some form in at least 30 jurisdictions; including Switzerland, the Netherlands, Belgium, Luxembourg, Colombia, Canada, Germany, Spain, Portugal, New Zealand, Austria, Ecuador, all six states of Australia, and in ten states and one district in the United States (**Figure 1**) (1). In Spain, Italy, Germany, Montana, Portugal, Columbia, and Ecuador, the practice has been deemed legal but comprehensive legislation has not yet been passed to regulate the practice. In most jurisdictions, there are various procedural safeguards in place to regulate access to lethal medications (15). **Table 2** provides a summary and comparison of the eligibility and safeguarding criteria of assisted dying legislation across the world. Please note, the information provided on the table reflects the current state of affairs up to April 2024 and will be subject to change.

**Table 2:** International eligibility criteria for assisted dying.

Jurisdiction	Legalized	Method	Key Criteria	Age	Residency
Legal on Basis of Terminal Prognosis					
United States^1^	1997	PAS	<6 mos to live	18+	Required, except Oregon & Vermont
Australia^2^	2019	Euthanasia & PAS	6-12 mos to live^3^	18+	Required
New Zealand	2021	Euthanasia & PAS	<6 mos to live	18+	Required
Legal on Basis of Irremediable Condition					
Netherlands	2002	Euthanasia & PAS	Without prospect of improvement; no reasonable alternative	12+ with parent consent; 16+ without parent consent	Not required
Belgium	2002	Euthanasia	Serious, incurable disorder; no reasonable alternative	Any age	Not required
Luxembourg	2009	Euthanasia & PAS	Incurable medical situation	18+	Not required
Canada	2016	PAS	Grievous and irremediable condition	18+	Required
Spain	2021	Euthanasia & PAS	Severe, chronic, debilitating condition or severe and incurable disease	18+	Required
Legal on Basis of Non-Selfish Motives					
Switzerland	1937	Assisted Suicide (no physician)	No selfish motives; typically performed by non-physicians	Any age	Not required
Legal, Not Yet Fully Regulated					
Montana (USA)	2009	PAS	Not established	18+	Not established
Colombia	2014	Euthanasia & PAS	Terminal illness and unbearable suffering	6+	Required
Italy	2019	Euthanasia & PAS	Grievous and irremediable medical condition causing enduring suffering	Not established	Required
Germany	2020	PAS	Not established	Not established	Not established
Portugal	2023	Euthanasia & PAS	Terminally ill, incurable condition, intolerable suffering	18+	Required
Ecuador	2024	Euthanasia	Not established	18+	Required

Informed by Hobbs and Gajjar (17) and Richardson (1).

1 Includes Oregon, California, Colorado, Hawaii, Maine, New Jersey, New Mexico, Washington, Vermont, Washington DC.

2 Includes Victoria, Western Australia, Tasmania, New South Wales, Queensland, South Australia.

3 In some states of Australia, persons with neurodegenerative disorders and less than 12 months to live are eligible.

### 1.3 Eligibility criteria

#### 1.3.1 Terminality

In most jurisdictions, a person must have a terminal illness to undergo assisted dying. The Benelux countries — including the Netherlands, Belgium, Luxembourg— are notable exceptions. In Canada, legislation initially restricted the practice to terminal conditions, but laws were later amended after court challenges to remove these criteria (19). In Switzerland, assisted death has been legal since 1942, and a terminal illness has never been required (18). Research suggests that 21-32% of people who die by assisted dying each year in the country do not have a fatal illness (20).

In jurisdictions that require a terminal illness (See **Table 2**), legislation rarely provides a comprehensive, clinically applicable explanation of how determinations of terminality should be made, and thus this criterion is subject to interpretation. In the United States, laws require physicians to deem with reasonable medical certainty that the patient will inevitably die within six months before prescribing assisted dying medications (20). But determining the prognosis of a chronic condition — even a physical one — is difficult in clinical practice (21). Studies suggest clinicians are routinely inconsistent, inaccurate, and imprecise in their prognostic estimations of life expectancy, with a tendency toward underestimation (22). Further research has shown that physicians tend to be pessimistic in their prognosis of outcomes in seriously ill patients, and factors such as the physician’s personality and attitudes have been shown to bias their prognosis (23). In the United States, laws grant immunity from civil and criminal liability for physicians who act under the law in “good faith” (24, 25). This provides protection to physicians but not to patients, who are at risk of greatest harm — the loss of life itself.

#### 1.3.2 Irremediability

In countries that permit assisted death for non-terminal conditions, statutes typically require the person has an irremediable condition causing unbearable suffering (**Table 2**). Switzerland is an exception; Swiss law only requires the person providing assistance in death not have selfish motives (1). In the Netherlands, the ‘unbearability’ of the patient’s suffering must be ‘*invoelbaar’*, or palpable to the physician (14, 26). Some countries stipulate that suffering must arise from a physical illness (e.g. Australia and Canada); whereas in Benelux countries, laws do not make a distinction between physical suffering and mental or emotional suffering (14).

Many scholars have highlighted the challenges these criteria pose in clinical practice. Specifically, the experience of suffering in general — and unbearable suffering in particular — can only be appraised by the individual experiencing it and its appraisal may be influenced by doctor-patient dynamics (10, 14, 27). Highlighting the ethical challenges these criteria pose for ED clinicians, Komrad and Hanson (14) state they: “opened the door to patients with psychiatric conditions, even those without additional medical conditions, to receive physician-assisted death, often with support from their treating psychiatrists who had been trying to prevent their suicide” (p. 183).

In addition to unbearable suffering, many laws further stipulate that the person’s condition must be *incurable* or *irremediable*. Canada’s statute defines an irremediable medical condition as one in which there is an advanced state or irreversible decline that causes enduring physical or psychological suffering (28). In the Netherlands, physicians and patients must agree that there is *no other reasonable alternative* to relieving the person’s suffering (29). Some scholars have raised concerns that in psychiatry, clinicians have little empirical basis to make prognostic predictions about irremediability (30). Others have noted that psychiatric suffering may be both a symptom of the illness and remediable over time (31). Critically, the Dutch law allows patients to refuse treatments they deem unacceptable, so even a person who has not received evidence-based treatment may qualify for assisted death under the law (14). In practice, the impact of these laws is substantial. A review of cases in the Netherlands found that 27% of patients with personality disorders who were euthanized had never received psychotherapy (10).

#### 1.3.3 Voluntary request

**Figure 2:**
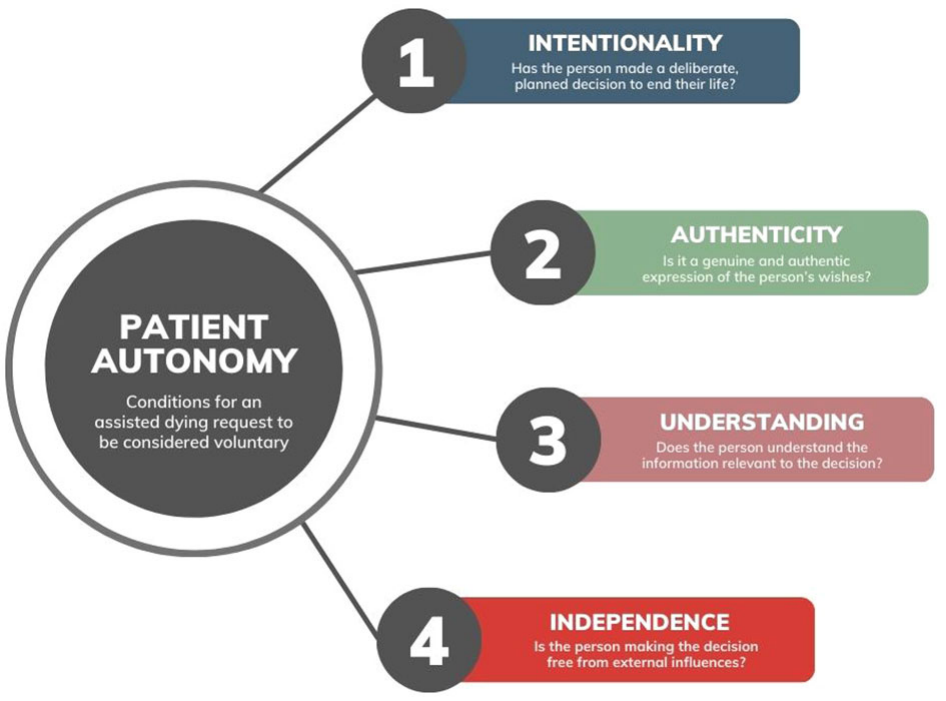
Conditions for a request to be considered voluntary in assisted dying decisions. Informed by Beauchamp and Childress (33), Marceta (34), and Sjöstrand and Juth (35).

In every jurisdiction where assisted dying is legal, it may only be carried out at a patient’s voluntary request. The concept of voluntary informed consent is traceable the Nuremberg Code, which emphasizes that a person should “be able to exercise free power of choice, without the intervention of any element of force, fraud, deceit, duress, overreaching, or other ulterior form of constraint or coercion” (32 p. 1). For a request to be considered voluntary, it must be made autonomously, which involves acting (1) with deliberate intention, (2) from authentic desires, (3) with sufficient understanding, and (4) free from controlling or coercive influences (**Figure 2**). Many countries state a request cannot be considered voluntary if it arises from a psychiatric disorder that impairs decision making (e.g. depression) or is made under pressure from others (e.g. health professionals, relatives, or the society in general) (29).

#### 1.3.4 Mental Capacity

For assisted death to be considered voluntary, the requesting individual must possess adequate *mental capacity* to decide to end their life (**Table 3**). Assessing mental capacity in a life-or-death decision is never straightforward, but it is particularly complex in patients with psychiatric illnesses, which can impair decision making capacity (27, 40). A person’s degree of mental capacity may fluctuate day to day with symptoms of illness (41), and clinician assessments may not catch subtly diminished capacity in individuals with complex psychiatric conditions (42, 43). If the person has a mental illness, a psychiatrist may be required to evaluate capacity before requests are granted (14). However, this requirement is not adequately enforced in some jurisdictions (14). For example, in Oregon, only three patients (less than 1%) were referred for psychiatric consultations in 2022 (44).

**Table 3:** Factors related to assessment of mental capacity in medical decision-making.

Decision Specific	Reflects patient’s ability to make a specific decision in a particular context; not generalizable between different types of decisions (e.g., treatment vs. assisted dying)
Variable	Acknowledges a patient may have intermittent capacity, which fluctuates with symptoms and severity of illness
Relative to Risk	Recognizes decisions involving greater likelihood and severity of risk require more evidence (and/or degree) of capacity
Exists on a Continuum	Acknowledges a patient may have diminished capacity; patient may be able to make decisions in some domains but not others

Informed by Appelbaum (36); Beauchamp and Childress (33); Berens and Kim (37); Lahey and Elwyn (38); Okai et al. (39).

Physicians are usually responsible for assessing capacity (14). While standardized tests for mental capacity exist, studies suggest clinicians rarely use formal assessments in clinical practice (45, 46). Studies have shown physicians frequently disagree on the competency of psychiatric patients who request assisted death — inter-rater reliability of capacity judgments by clinicians without the aid of standardized assessments is low, and modest evidence suggests clinicians tend to overestimate capacity (6, 47–50). Research has also shown that the assessing physicians’ own personal values and opinions may bias their judgments of a patient’s mental capacity (51).

There are currently no mental capacity assessment tools that have been validated specifically for assisted dying. In the context of healthcare decisions, four criteria developed by Appelbaum and Grisso (52) are widely used. They include the ability to (1) *understand* the relevant information as it relates to oneself, (2) *appreciate* the situation and its consequences, (3) *reason* about different treatment options, and (4) *communicate* a choice (52). When mental illness impairs capacity, it most frequently affects the “appreciation” dimension, which reflects a person’s ability to apply the consequences of a decision to themselves (53). The Mental Capacity Act (2005) has been discussed internationally as an established legal framework for assessing mental capacity in treatment decisions (54). Although it is not applicable to assisted dying under current UK law, it has influenced similar legislation in other countries (55).

Mental capacity is often discussed in terms of thresholds. Some conceptions hold that the more severe the consequences of a medical decision, the higher the threshold for mental capacity needs to be (56–58). While there is some debate about whether risky decisions also require a higher *degree* of capacity; most scholars agree that at minimum, decisions involving greater risk to life require a higher *threshold of evidence* of adequate mental capacity (33, 37, 59, 60).

In a practical sense, this means patients may be competent to make decisions in some domains, but not others. When a serious risk — such as death — is present, then a higher standard of evidence for mental capacity may be required. For example, a patient may be found to have capacity to refuse treatment (e.g., hospitalization or administration of psychotropic medications) but not to authorize their own death through assisted dying. This approach recognizes the dire consequences of a false positive in a life-or-death decision – if a clinician misjudges the patient’s capacity in an assisted dying decision, the consequences are irreversible.

### 1.4 Expansion of legislation

**Figure 3:**
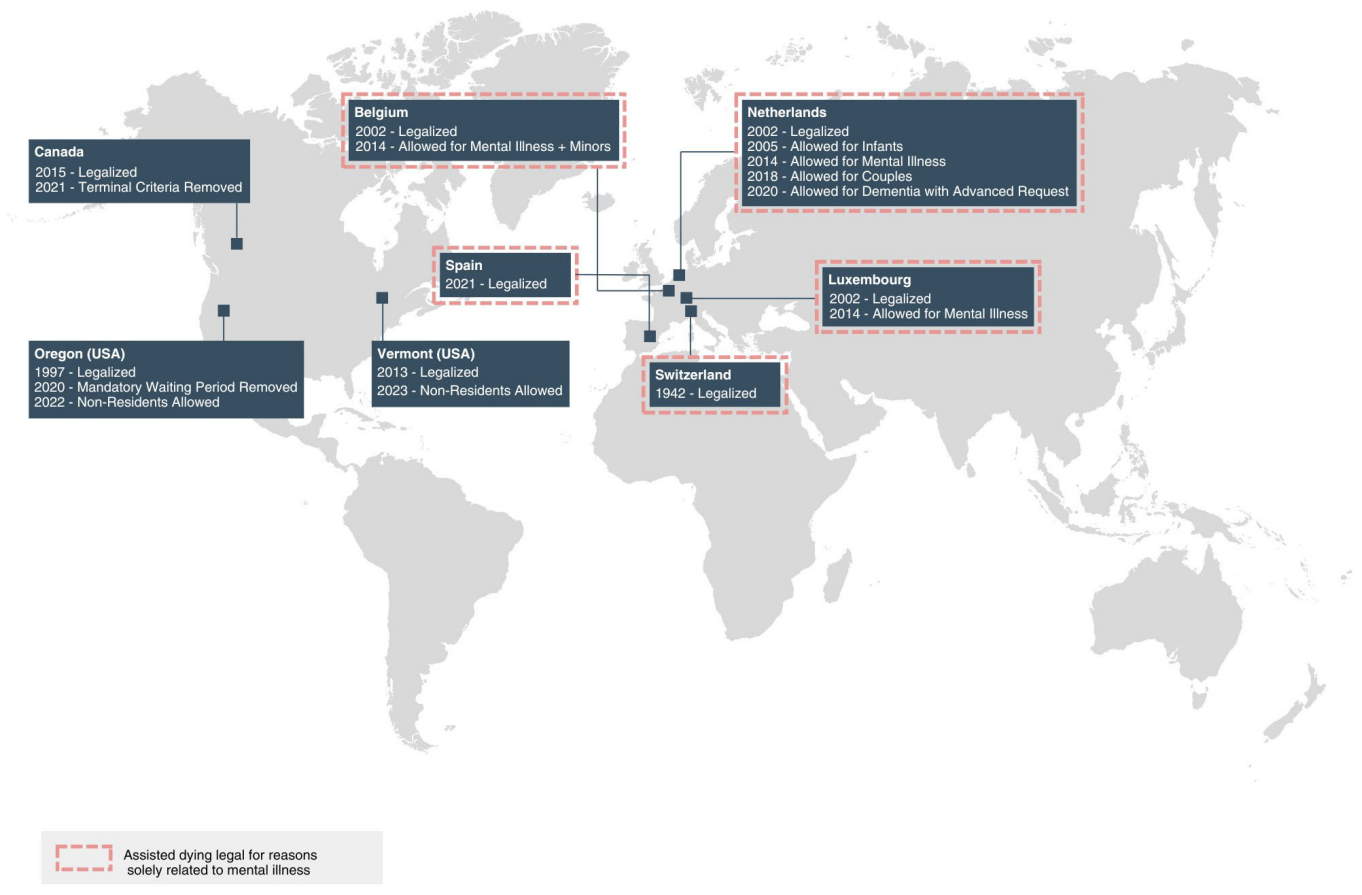
Expansion of assisted dying legislation through amendments and court challenges. Informed by Mroz et al. (15), Bartsch et al. (18), Carter v. Canada (61), Raus (62), Verhagen and Sauer (63), Gauthier et al. (64), Dresser (65), Vermont Department of Health (66), and Oregon Medical Board (67).

In some jurisdictions, statutes with initially stringent eligibility criteria have been expanded through amendments and judicial challenges (61). The two most common ways this has occurred relevant to EDs pertain to age and residency (**Figure 3**).

#### 1.4.1 Age

The Netherlands, Belgium, Switzerland, and Colombia allow assisted death for minors (**Table 2**). In Belgium, the practice was initially restricted to adults, but legislation was expanded in 2014 to remove age restrictions (62). Theoretically, children can request euthanasia at any age, provided they meet the other eligibility criteria (62). In the Netherlands, eligible children have been able to request euthanasia from the age of 12 since the law’s inception (68). Sixteen- and seventeen-year-olds do not need parental consent, but parents must be involved in the decision-making process (68). In 2005, the Netherlands legalized euthanasia for infants through the Groningen Protocol (63). A study of death certificates in the Netherlands found that 2.7% of all deaths of Dutch children involve assisted dying, and most occurred at the explicit request of the parents (2% out of 2.7%) (69). The capacity of children to consent to end their lives and the role of parental consent is a highly controversial and contested issue (70–72).

#### 1.4.2 Residency

Some countries and states have expanded their laws to allow non-residents to undergo assisted dying (**Table 2**). This has led to the growth of what has colloquially been called “suicide tourism,” or the practice of traveling to a jurisdiction where assisted dying is legal to end one’s own life (64). Non-residents can legally obtain lethal medications to end their life in Switzerland, Belgium, and the Netherlands; in Switzerland, nearly half of all people who died through assisted suicide are non-residents (18). In 2023, Vermont and Oregon passed amendments to allow physicians to legally prescribe MAiD to non-residents, after settlement agreements were reached in two lawsuits brought by the lobbying group Compassion & Choices (24, 65, 66). Both states allow MAiD to be prescribed via Telemedicine, although physicians may be subject to civil and criminal liability for prescribing lethal drugs if patients are not physically located in the state at any stage of the process (66, 67). It is not clear whether physicians would be liable to criminal or civil prosecution if out-of-state patients return to states where assisted dying is not legal to ingest the medications. In at least one case involving a person with an ED, a Colorado-based physician prescribed MAiD across state lines to a patient in California (2).

### 1.5 Inadequate reporting and failure of safeguards

While reporting is mandatory in all jurisdictions, publicly available reports rarely include detailed information (e.g. psychiatric diagnoses) about the characteristics of patients who underwent assisted death (73). This paucity of information has made it difficult for researchers and lawmakers to assess the adequacy of current safeguards and the rates of assisted death in patients with psychiatric disorders (73). Out of 27 jurisdictions where assisted dying is legal, a recent study found only 16 regularly published reports, and most only provide limited demographic data (73). Only two jurisdictions identify the number of patients referred for psychiatric evaluation, and none provide information about psychiatric diagnoses at the time of patients’ requests (73). It is therefore not possible to determine how many patients with EDs have undergone assisted death internationally because data for the vast majority of assisted death cases are not available for public inspection (73). **Box 1** provides an overview of the reporting practices in each jurisdiction.

Box 1 **Reporting practices in countries allowing assisted death for mental disorders.**

**Belgium:** The Federal Committee for Evaluation and Control (FCCE) in Belgium is responsible for oversight of euthanasia. It publishes biannual quantitative reports, which sometimes include broad categories of psychiatric diagnoses (e.g. mood disorders, developmental disorders, and stress-related disorders) of patients who underwent euthanasia for solely psychiatric reasons (74). Detailed case summaries are not made public, but they may be obtained (in French or Dutch) for academic research purposes in response to a substantiated request to the FCCE (11).

**Luxembourg:** Luxembourg’s National Commission for Control and Evaluation (NCCE) publishes biennial quantitative reports, which include statistical and demographic data. While psychiatric diagnoses are not reported, assisted deaths remain relatively low overall (e.g., 34 in 2023) (75), and the most recent report stated: “The Commission notes that until now no euthanasia has yet been carried out for people with mental disorders” (69 p. 22).

**The Netherlands:** The Regional Euthanasia Review Committees (RTE) are tasked with scrutinizing physician-submitted reports of assisted death in the Netherlands. They publish annual quantitative reports and select 1-2% of cases to publish as qualitative case summaries each year (in Dutch), to illustrate to physicians and the wider public how the committees apply and interpret eligibility (“due care”) criteria (76). Critically, the committees review reports after the death has already occurred, which means transgressions can only be identified retrospectively.

**Switzerland:** In Switzerland, which has the least restrictive laws regarding assisted dying and mental illness, there is no central registry for reporting assisted suicide (77). The Swiss Federal Statistical Office has worked with assisted suicide organizations to document deaths as a separate category on death certificates since 2011 (18). However, these records only contain limited information about the persons who underwent assisted suicide and do not usually specify psychiatric diagnoses (18, 20, 64).

**United States:** Ten of the eleven states in which assisted dying is legal publish annual quantitative reports. Each state varies substantially in its reporting practices; some states (e.g., Colorado, Hawaii, and Maine) report on the number of patients who receive MAiD prescriptions but not on how many patients died (73). There does not appear to be a mechanism for tracking how many unused MAiD prescriptions are circulating in the community (73). Oregon is the only state that reports the number of patients referred for psychiatric evaluation, and no states report on psychiatric diagnoses (73).

#### 1.5.1 Evidence of Inadequate Safeguards

Some evidence suggests that reporting requirements and safeguarding criteria are sometimes ignored and transgressed, especially in jurisdictions where transgressions are not always prosecuted (33, 78–80). Smets et al. (79) found that half of assisted deaths in Belgium went unreported by physicians, and eligibility criteria were not met more frequently in unreported cases than in reported cases (88% vs 18%, respectively). The majority of unreported cases (92%) involved acts of euthanasia that the consulting physician did not perceive to be euthanasia (79). In the Netherlands, research suggests that approximately 20% of euthanasia cases are not reported to the review committees, down from 46% in 2001 (78, 81, 82).

### 1.6 Assisted dying for EDs

The first reports of assisted death in patients with EDs in the peer-reviewed literature emerged from Belgium and the Netherlands (6, 12). In a review of patients euthanized for psychiatric disorders in Belgium between 2007 and 2011, Thienpont et al. (12) noted ten patients out of 100 were diagnosed with EDs. Doernberg et al. (6) identified four patients with EDs who were euthanized in the Netherlands between 2011 and 2014. Scholars have suggested that the actual number of patients with EDs euthanized in these countries during these time periods is likely much higher because the review committees only publish detailed case information (e.g. inclusive of psychiatric diagnoses) for 1.5% of all reports of euthanasia (14, 76).

Assisted death for reasons solely related to a mental disorder is legally permitted in Switzerland, the Netherlands, Belgium, Luxembourg, Spain, and Austria (**Table 2**). In the Netherlands, euthanasia has been performed on patients with intellectual disabilities, autism spectrum disorder, schizophrenia, and a wide range of psychiatric conditions (10, 76, 77). In Spain and Austria, the practice was only recently legalized, and comprehensive regulations have not yet been put in place (1). In 2019, Canada passed legislation that would amend its existing law and allow MAiD in those with unbearable psychological suffering caused by psychiatric illness. As of the date of publication, this component of the bill has been repeatedly delayed and not yet come into effect. In the United States, there are increasing calls for laws to be amended to allow MAiD for mental illness on the grounds of parity (30, 83).

### 1.7 Assisted death in EDs and lack of research

Although there have been a flurry of commentaries on recent reports of patients with EDs who were prescribed MAiD, there has been no systematic effort to identify and aggregate known cases of assisted death in patients with EDs internationally (3, 4, 84–90). Further, little is known about the clinical rationales physicians have used to justify assisted death in these patients. This systematic review aims to aggregate known cases of assisted death in patients with EDs, identify the jurisdictions in which they have occurred, and systematically review the clinical rationales which have been used to justify assisted death in these cases.

There are several contributions this study makes to the literature. First, it is the first study to systematically review all known cases of assisted dying in patients with eating disorders across both peer-reviewed studies and official government reports. Second, it describes the clinical reasoning that has been used to affirm eligibility and grant patients’ requests, identifying and clarifying concepts lacking validity and empirical support. Finally, it establishes a framework for future research on the evidence for these rationales and highlights substantial gaps in the reporting of assisted deaths in patients with psychiatric disorders, which may guide both policy decisions and further research.

## 2 Methodology

Using methodology adapted from (9), we performed a systematic review of studies and reports describing patients with EDs who underwent assisted dying. Our aims were two-fold: (1) identify reports of patients with EDs who underwent assisted death and the jurisdictions in which they occurred, and (2) describe the clinical rationales that were used to affirm eligibility and justify its use in these patients.

### 2.1 Reflexivity

Reflexivity is a process that documents the researchers’ awareness of their own biases, assumptions, and perspectives and can help reduce the influences of these biases on the research process itself (91). Of relevance for this review, CR is the Executive Director of a nonprofit that helps individuals recover from EDs. She has worked with patients at all stages of treatment, from inpatient hospitalization to community care. As a teenager, she was hospitalized for anorexia with a BMI in the single digits and compelled to undergo court-mandated treatment. She has been recovered for nineteen years. CCC is a professor and licensed psychologist. She treats individuals with disordered eating and conducts research on the prevention and treatment of EDs and trauma. She has recovered from an ED for which she received outpatient care.

## 3 Search strategy

A systematic search of the literature was carried out using the following databases: PubMed, PsycINFO, Web of Science, and Academic Search Complete (EBSCO). To ensure that the main search query was comprehensive and balanced in terms of sensitivity, several preliminary iterations of the search were conducted using different terms and filters. The final search queries (**Supplementary Material 1**) were reviewed and approved by a medical librarian at University at Buffalo.

The search was performed in January 2024 and updated in May 2024. Both authors independently searched and used the snowball method to identify studies not detected in the database searches. Results were aggregated using Covidence software to remove the duplicates among the databases, and each reviewer evaluated all titles and abstracts to identify studies that met the inclusion criteria.

Given the dearth of descriptive case reports involving persons with EDs in the peer-reviewed literature, publicly available government reports were reviewed in all jurisdictions where assisted dying is legal for persons with psychiatric illness (Belgium, the Netherlands, Switzerland, and Luxembourg) as of April 2024. While MAiD is not legal for suffering caused by psychiatric illness in the United States, some cases have been reported there, so reports from all eleven jurisdictions where assisted dying is legal in the United States were also included (2). For a description of the reporting practices in each jurisdiction, please see **Box 1**.

The Netherlands is the only jurisdiction that also publishes a select number of descriptive case reports (in Dutch) on its website. To identify case reports describing persons with EDs who underwent euthanasia, we replicated a search strategy reported on by Kim et al. (8), Nicolini et al. (10), and Tuffrey-Wijne et al. (76). Specifically, we identified all published case reports of persons who underwent euthanasia on the Dutch RTE website, then filtered the cases by those involving psychiatric disorders. We then translated and read all cases to identify those involving patients with EDs.

### 3.1 Inclusion and exclusion criteria

We included studies and case reports that met the following criteria (1): reported on a case in which at least one person with an ED underwent assisted dying (2), published after 2000. This time frame was chosen as it aligns with the legalization of assisted dying in many jurisdictions (15), and (3) reported in a peer reviewed journal or in a formal government report.

**Figure 4:**
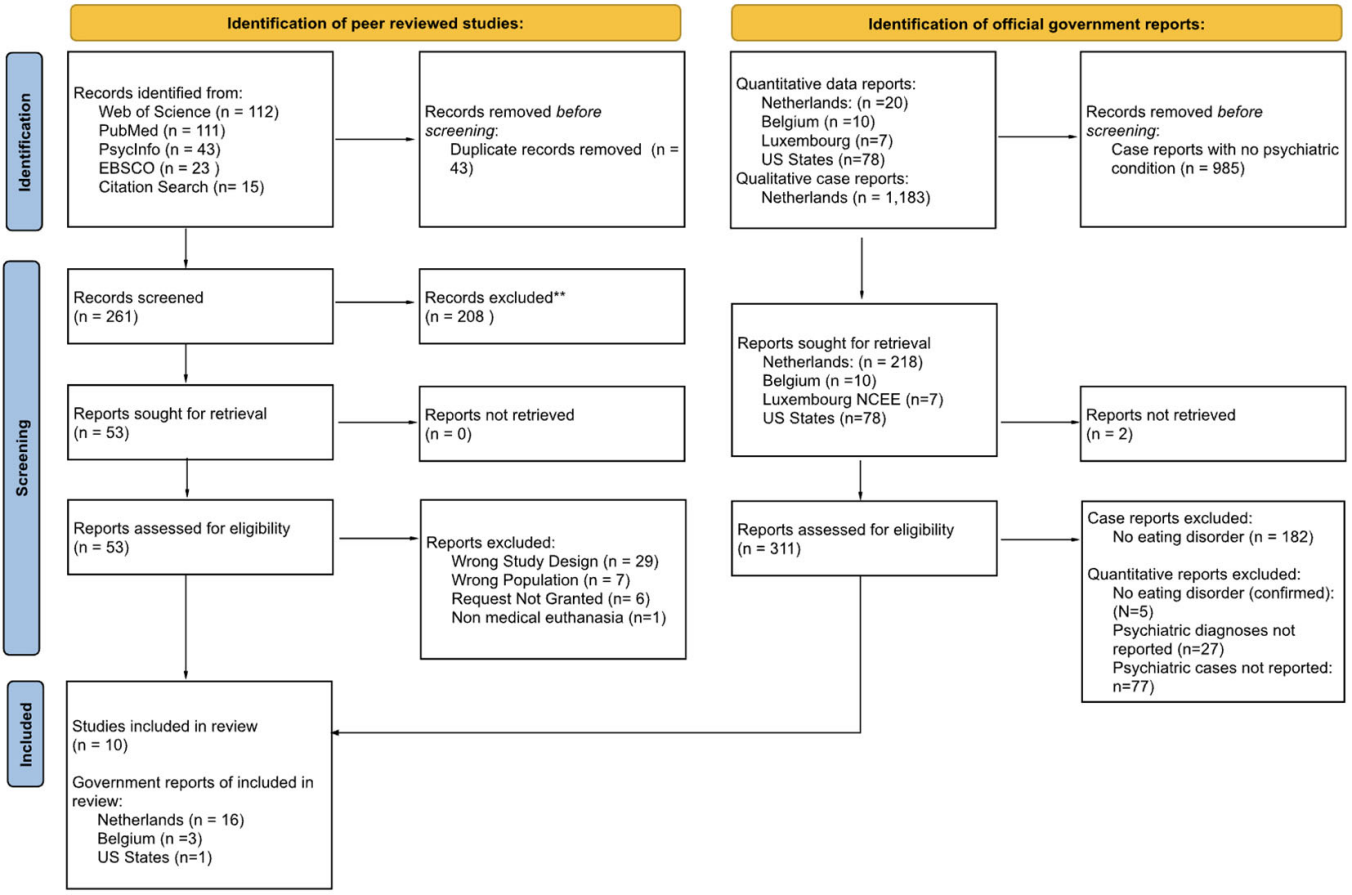
PRISMA flow chart of studies and reports identified in review. **Exclusion reasons included wrong population (e.g. non-human animals) and wrong condition (e.g. cancer, kidney disease, other psychiatric disorders).

Our search conditions excluded commentaries, theoretical articles, response articles, and articles about the ethics or legal framework for assisted dying which did not describe specific cases (**Figure 4**). We also excluded papers that referred to anorexia as a medical term (loss of appetite) and articles that reported on cases of “passive euthanasia” (withholding treatment or allowing a patient who refuses treatment to die of starvation).

### 3.2 Study selection and data extraction

**Figure 5:**
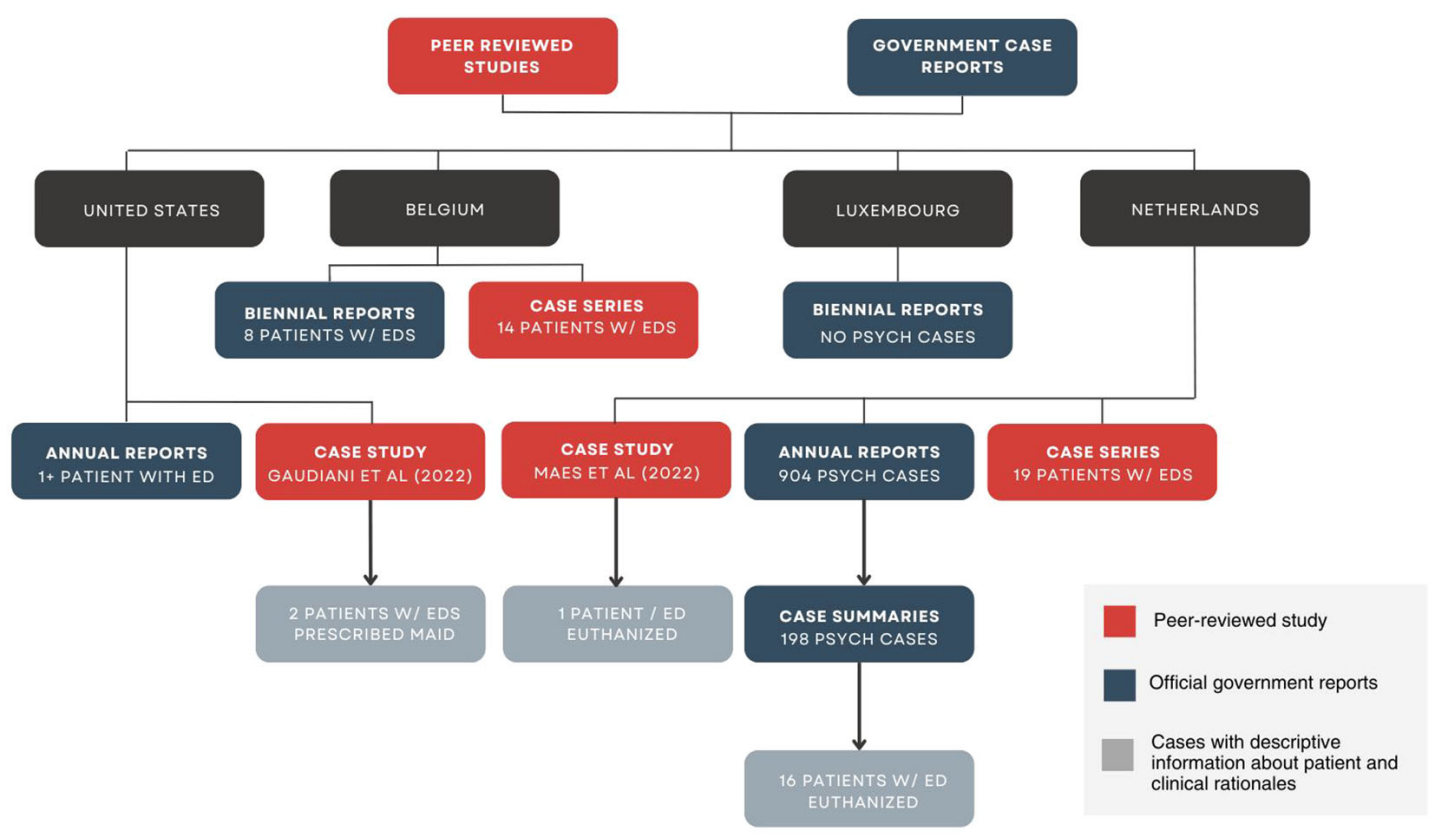
Identification of studies and reports with clinical rationales and patient characteristics.

Both authors independently screened the titles and abstracts of all articles using the criteria described above. Discrepancies were resolved through discussion. Dutch articles were initially translated using two translation softwares (ChatGPT Plus, then cross-referenced with Google Translate) and checked for accuracy by a native Dutch speaker who is a clinician in EDs. Full text screening for English articles was performed by both co-authors, and screening for Dutch case reports was also performed by the third Dutch-speaking reader (**Figure 5**).

To identify the clinical rationales used to justify the use of assisted dying in each case, we performed a content analysis adapted from a method described by Kim et al. (8). First, we referred to the legal framework for assisted dying (**Table 2**) to identify three key eligibility criteria that are common across multiple jurisdictions (terminality, irremediability, and voluntary request). Second, we developed a coding scheme for rationales within each domain iteratively by reading each case report and repeatedly comparing clinical rationales referenced in each report against the key criteria domains. One author read all the reports and developed the coding system, and the second author confirmed the coding system by reading through the reports again. Discrepancies were resolved through discussion. Finally, the native Dutch reader read the reports and confirmed the accuracy of the coding results against the reports in Dutch.

## 4 Results

We identified 10 peer-reviewed articles and 20 government reports describing at least 60 patients with EDs who underwent assisted dying between 2012 and 2024 (**Table 4**). Note that this figure does not represent the total number of patients with EDs who have undergone assisted dying in countries where it is legal. It represents only those which were identifiable via the limited data available in public reports. Of these 60 cases, we identified descriptive case summaries for 19 patients: 17 underwent euthanasia in the Netherlands, and 2 were prescribed MAiD in the United States. We were only able to extract clinical rationales in reports with descriptive case summaries; the other reports only provided limited quantitative data about the cases. A description of the study characteristics, patients described in each case report, and clinical rationales extracted from each report is included below.

**Table 4:** Characteristics of studies and reports included in review.

Studies and Government Reports with Case Descriptions			
Author (Year), Type	Sample	Country	Main Findings
Gaudiani et al. (2) *Case Study*	3 total, 2 prescribed MAiD	USA	Describes three deceased patients with anorexia; two were prescribed MAiD. Authors propose a new clinical category for “terminal anorexia.”
Maes et al. (92) *Case Study*	1 patient with ED	Netherlands	Describes two patients who donated organs after euthanasia. Authors suggest organ donation has minimal burden and may be helpful for families.
Dutch RTE (2013-2024) *Government Case Summaries*	16 patients with EDs	Netherlands	Cases are described to illustrate to physicians and the public how the legal criteria for euthanasia are applied.
Reviews and Case Series (No Case Descriptions)			
Calati et al. (77) *Systematic Review*	Review, N/A	International	Reviewed 24 studies of psychiatric euthanasia in the Netherlands, Belgium, and Switzerland.
Dierickx et al. (11) *Qualitative Analysis of Cases, 2002-2012*	179 total, 4 with EDs	Belgium	Euthanasia in patients with psychiatric disorders in Belgium gradually increased between 2002 and 2013, particularly in patients with mood disorders.
Doernberg et al. (6)^1^ *Qualitative Analysis of Cases, 2011-2014*	66 Total, 4 with EDs	Netherlands	Most physicians did not appear to use a high threshold to assess whether psychiatric patients had decision-making capacity to request euthanasia.
Kim et al. (8)^1^ *Qualitative Analysis of Cases, 2011-2014*	66 Total, 4 with EDs	Netherlands	Most psychiatric patients requesting euthanasia have chronic, severe conditions (e.g. autism, psychosis, EDs, PTSD) with a history of attempted suicide.
Kammeraat et al. (7) *Review of Medical Records 2012-2018*	154 total, 4 with EDs	Netherlands	In a review of researcher-selected records from a Dutch euthanasia center, most psychiatric patients requesting euthanasia were single females living alone with depression and multiple comorbid diagnoses.
Nicolini et al. (10)^1^ *Qualitative Analysis of Cases, 2011-2017*	74 total, 11 with EDs	Netherlands	Among persons with personality disorders who underwent euthanasia, 28% had not tried psychotherapy. In 50% of cases, the euthanizing physician was new to them and 36% had no psychiatrist.
Thienpont et al. (12) *Retrospective Descriptive Study, 2007-2011*	100 total, 10 with EDs	Belgium	Among 100 psychiatric patients who requested euthanasia, 50% had a personality disorder and 12% had autism. Includes one patient w/ “terminal anorexia,” who died by palliative sedation.
van Veen et al, 2019^1^ *Qualitative Analysis of Cases, 2015-2017*	35 total, 7 with EDs	Netherlands	Among 35 psychiatric patients euthanized for psychiatric disorders, 77% were women and 20% had ED diagnoses.
Quantitative Government Reports (No Case Descriptions)			
Oregon Death with Dignity Act Data Summary 2021	1+ with EDs	Oregon	Annual report states that 7 people died of “other illnesses.” Anorexia is listed under other illnesses, but the exact figure is not clear.
FCCE Reports (2022, 2016, 2014)	8 patients with EDs	Belgium	Persons with EDs underwent euthanasia in 2012 (N= 3), 2013 (N=2), 2014 (N=1), 2020 (N=1), 2021 (N=1).

1 Kim et al (2016) and Doernburg et al (2016) both studied the same sample of cases. Doernburg et al (2016), Nicolini et al (2019), and van Veen (2019) had some overlap in samples (6 patients).

### 4.1 Description of peer-reviewed studies

Of the 10 peer-reviewed studies, three study designs were represented: one systematic review, seven case series, and two case studies. A systematic review conducted by Calati et al. (77) aggregated all available data on psychiatric patients who have requested or undergone assisted dying between 2002 and 2020. We identified seven analyses of psychiatric euthanasia cases that included ED patients: five in the Netherlands, and two in Belgium (**Table 4**). There was some overlap in the samples of patients in four of the Dutch case series, noted in **Table 4**. ED patients represented between 2.2% and 20.0% of all psychiatric cases in these studies. Kammeraat et al. and Dierickx et al. reported significantly lower rates of EDs (2.6%; 2.2%) compared to those reported in other studies (6.1%, 10%, 14.9%, 20%; **Table 4**). Very few studies have been published on cases of psychiatric euthanasia in Switzerland, and those that exist often do not specify the psychiatric diagnoses of patients who underwent assisted suicide (18, 20, 64, 93). Therefore, it was not possible to confirm or rule out cases of assisted suicide in patients with EDs in Switzerland.

One case study has been published in the United States (2), which described three patients, two of whom were prescribed MAiD — one in Colorado, and another who resided in California and received a prescription from a Colorado-based physician. We found one additional peer-reviewed case study which described a patient with an ED in the Netherlands who donated her organs after euthanasia (92).

### 4.2 Description of government reports

We identified two main types of government reports: (1) quantitative reports, which included limited demographic data about patients who underwent assisted dying and (2) case summaries, which included more detailed qualitative information about individual patients and the circumstances that led to their death. In total, there were four quantitative data reports and sixteen government case summaries describing patients with EDs who underwent assisted death: sixteen from the Netherlands, one from the United States (Oregon), and three from Belgium (**Table 4**). The level of detail in each report varied widely between countries, and there were even significant inconsistencies in how deaths were reported in a single jurisdiction from one year to the next. In most reports, it was not possible to determine whether the report included persons with EDs, because psychiatric diagnoses of patients were not reported.

#### 4.2.1 Europe

In Switzerland, no government data reports were available. Of the five biennial reports from Luxembourg, there were no cases of psychiatric euthanasia reported (75). In Belgium, eight patients with eating disorders were identified in three data reports (94–96). Seven of the ten Belgian reports did not provide descriptive enough data to determine specific psychiatric diagnoses, so it was not possible to confirm or rule out whether patients with EDs were euthanized in these years (97).

Twenty quantitative data reports were available from the Netherlands, which listed 904 cases of psychiatric euthanasia. These reports did not report specific psychiatric diagnoses. However, 22% (*N* = 198) of psychiatric cases were published as detailed case summaries with information about the patients’ conditions and the circumstances that led to euthanasia. Of these, 8% (*N* = 16) described patients with EDs. For the remaining 78% of psychiatric cases (*N* = 706), it was not possible to determine the psychiatric diagnoses and thus cases involving patients with EDs could not be confirmed or ruled out.

#### 4.2.2 United States

Of the 78 data reports reviewed from U.S. states where assisted dying is *only legal for terminal conditions*, which report on a total of 11,983 cases, none include reporting on psychiatric conditions. Only one report mentions an ED specifically (44). Oregon’s Death with Dignity Report (44) noted that seven individuals were prescribed MAiD for *Other Illnesses*, of which anorexia was listed as an example condition in the footnote. It is unclear how many of those seven deaths were persons with anorexia. The category *Other Illnesses* appeared frequently across the 77 reports, and reports rarely provided examples of diagnoses included under this category (66). Thus, it was not possible to determine the number of patients with EDs who have died through MAiD in the United States.

There were no mentions of EDs in Colorado and California’s reports, despite cases involving patients with EDs having been previously reported in the literature (2). In the authors’ correspondence with Colorado’s Vital Statistics Program, an official confirmed that anorexia “has been reported by name as a terminal illness/condition and is presently counted among the ‘other illnesses/conditions’ in our reports” (98). Due to patient confidentiality requirements, the official was not able to confirm the number of cases or the years in which these were reported. The official noted a growing number of cases for which the terminal condition was identified as ‘severe protein calorie malnutrition.’ Twelve cases were reported between 2021 and 2023 — including nine in 2023 alone — compared to zero cases in previous years (99).

### 4.3 Characteristics of patients with EDs who underwent assisted death

Of the 60+ cases identified across all studies and reports, 19 included descriptive case summaries with information about the patients and the clinical rationales that were used to justify assisted death (**Figure 4**). All 19 patients were women (**Table 5**). Specifically, 32% were under the age of 30 (*N* = 6), 37% were between the ages of 30 and 50 (*N* = 7), and 31% were over 50 years old (*N* = 6). 61% (*N* = 11) had been diagnosed with anorexia, one person was described as obese (but her ED was not specified), and 28% (*N* = 5) had EDs (but the specific diagnoses were not identified).

**Table 5:** Characteristics of ED patients who underwent assisted death.

Characteristic	No.	Percent
Women	18	100%
Age group		
18-30	6	32%
30-40	6	32%
40-50	1	5%
50-60	1	5%
60+	5	26%
Type of ED		
Anorexia nervosa	12	63%
Not specified	7	37%
Underweight/Malnourished	13	68%
Suicidal symptoms		
Suicidal thoughts	11	58%
Multiple suicide attempts	7	37%
Depression and anxiety		
Depressive Symptoms	17	89%
Anxiety Symptoms	12	63%
Trauma symptoms		
Dissociative Symptoms	4	21%
Flashbacks/Nightmares	5	26%
Other symptoms		
Psychotic Symptoms	3	16%
Self-injury	6	32%
Poor Social Functioning	11	58%
Substance Use	2	11%
Physical Disability	5	26%

All but one person described in the case reports had multiple comorbid psychiatric diagnoses. Rates of comorbidity were high; 95% had more than one psychiatric disorder, 61% had more than three, and nearly a quarter had four or more comorbid conditions (**Table 6**). Specifically, obsessive compulsive disorder (OCD) and post-traumatic stress disorder (PTSD) were common, occurring in 33% and 37% of cases respectively. One patient was described as having a mild intellectual disability, and 16% of patients (*N* = 3) had autism spectrum disorder. Nearly half of patients were diagnosed with at least one personality disorder.

**Table 6:** Psychiatric comorbidities in ED patients who underwent assisted death.

Diagnosis	No.	Percent
ED	19	100%
PTSD	7	37%
Depressive disorder	10	52%
Obsessive compulsive disorder	6	32%
Autism spectrum disorder	3	16%
Personality disorder	9	48%
Borderline personality disorder	5	26%
Avoidant personality disorder	1	5%
Other personality disorders	4	21%
Schizoaffective disorder	1	5%
Conversion disorder	2	11%
Multiple comorbid conditions	18	95%
3+ comorbid conditions	11	61%
4+ comorbid conditions	4	22%

Notably, 58% of patients were described as chronically suicidal, with 37% having made multiple past suicide attempts. Depression was extremely common; 89% of the patients were described as depressed, and over half were described as having poor social functioning. Self-injury (32%), psychotic symptoms (16%), dissociation (11%), and substance abuse (11%) were also reported in many patients. More than a quarter of patients were described as experiencing flashbacks and nightmares, and 63% reported symptoms of anxiety.

### 4.4 Clinical rationales

We identified 18 clinical rationales used to justify assisted death for patients with EDs (See **Table 7**). We categorized rationales into three content domains based on the most common legal criteria for assisted dying internationally: (1) terminality, (2) irremediability (condition and suffering), and (3) voluntary request. Exemplary quotes representing each rationale are provided in **Table 8**. Other notable features of the rationales are listed in **Table 9**.

**Table 7:** Identification of clinical rationales used in assisted dying for EDs.

Domain	Rationales	No.	Percent
Irremediability			
	Patient’s suffering is unbearable.	18	95%
	Patient’s condition is hopeless.	17	89%
	Patient has a severe and chronic form of illness.	9	47%
	Patient has been ill for a long time.	18	95%
	Patient’s prognosis is poor.	17	89%
	Treatment has produced no lasting results.	17	89%
	There are no realistic treatment options.	17	89%
	Patient has a treatment-resistant condition.	8	42%
	Healing/cure is no longer possible.	9	47%
	Further treatment would be futile.	2	11%
Terminality			
	Patient has a terminal condition.	2	11%
	The patient’s death is imminent.	2	11%
	Patient is at a terminal stage.	2	11%
Voluntary Request			
	Patient has decision-making capacity.	18	95%
	Patient has been adequately informed.	16	84%
	The patient’s wish to die is well considered.	18	95%
	The patient has made a consistent request to die.	19	100%
	Patient choice/autonomy must be respected.	10	53%
	The wish to die is not a symptom of mental illness.	5	26%

**Table 8:** Example quotes from clinical rationales used in cases of assisted death in patients with EDs.

Article Patient Characteristics	Terminality Domain	Irremediability Domain	Voluntary Request Domain
United States			
Gaudiani et al. (2) Female, 36 yrs, AN purging subtype Oregon, United States	“Dr. G completed the MAiD forms as consulting physician, given that Jessica’s prognosis was presumed to be *6 months or less*.”	“Dr. G spoke with Jessica’s parents repeatedly, assuring them that guardianship and forced treatment were likely now to be *futile.*” “Suffering from *unrelenting and irredeemable disorders*, these patients made difficult choices, ultimately deciding “enough is enough”	“Jessica waited several weeks to fill the MAiD prescription. She then set multiple dates to use it over a couple of months and *changed her mind* as that date got closer.” “She repeatedly told her family that *she didn’t want to die …* but she just couldn’t continue to exist this way.”
Gaudiani et al. (2) Female, 36 yrs, AN restricting subtype, OCD, depression California, United States	“Given her faster metabolism, if Alyssa abandoned her attempts to consume a higher meal plan, she would clearly have a *less than six-month prognosis.”* “MAiD [was] not pursued in isolation, but rather in the context of being in hospice care following a *terminal diagnosis* of anorexia (i.e., estimated 6 months or left to live).”	“Alyssa’s parents asked whether any treatments remained that might yet change the outcome of her course, specifically noting that Alyssa *had not completed* a full residential eating disorder program, never fully restored weight, and hadn’t had a feeding tube.” “Dr. G noted that if someone restricts the “tube God gave them,” i.e. their esophagus, *they would also be very likely* to restrict [their food] through a surgical feeding tube, so *that would not be a long-term solution*.”	“After a local psychiatrist confirmed that *Alyssa clearly possessed decision-making capacity*, the palliative care doctor fully accepted Alyssa’s right to enter home hospice care … however *he ultimately felt personally unable* to write the MAiD medication prescription.”

	Irremediability Domain		Voluntary Request Domain
The Netherlands			
Dutch RTE, 2024-016 Female, 60-70 years old, binge eating disorder	“The doctor was also convinced that the patient’s suffering was unbearable and, according to prevailing medical opinion, *without prospect of improvement.*” “According to the consultant, there were *no longer any reasonable treatment options* for the patient’s situation.”		‘The doctor deemed the patient decisionally capable with regard to her euthanasia request.’
Dutch RTE, 2023-067 Female, 20-30, anorexia nervosa	“Even though [the psychiatrist] noted that *there were no realistic treatment options specifically aimed at curing the depressive disorder or eating disorder*, the independent psychiatrist gave two pieces of advice to possibly improve the quality of life.” “*The chance of recovery was estimated as small* and it was disproportionate to the *great burden* on the patient.”		“The patient was decisionally capable. There was no indication that there were any internal or external factors that could possibly have influenced the patient’s choice.” “She was able to weigh diagnostic information well and translate it to her own situation and had the ability to weigh the information.”
Dutch RTE, 2023-034 Female, 20-30, AN, autism, PTSD, BPD, neurological disorder	“The independent psychiatrist saw indications of an ASD but considered the *chance of effective treatment to be low*. Due to *the absence of a chance of treatment* for the ASD, the independent psychiatrist was of the opinion that any treatment of ASD should not be made a condition of the euthanasia process.”		“The patient could not benefit from psychotherapy due to a *lack of reflective and mentalizing capacity*, in combination with rapidly increasing tension that caused dissociation and withdrawal.”
Dutch RTE, 2023-004 Female, 18-30, eating disorder, autism	“Treatment aimed at autism spectrum disorder was not used, because that was not the biggest problem and because ASD *cannot be treated in a therapeutic sense.”* “The patient suffered greatly from her *overpowering and disabling* obsessive compulsive disorder.”		“She did not appreciate that her life was saved by the doctors.” “The conclusion was that there was an indication for palliative care, that *the patient was decisionally capable* to ‘stop eating and drinking.’”
Dutch RTE, 2023-057 Female, 40-50, anorexia, schizoaffective disorder	“She had been seriously underweight *for years.*” “She had *no hope* for recovery or improvement.” “The doctor was also convinced that the patient’s suffering was unbearable and, *according to prevailing medical opinion, without prospect of improvement*.”		“She was *chronically depressed*, which caused her to *constantly struggle with suicidal thoughts* and express this by *attempting suicide.”* “The patient was able to put *with great clarity* why she made the request.”
The Netherlands			
Dutch RTE, 2022-085 Female, 20-30, anorexia, autism, mild intellectual disability, OCD, PTSD, suicidality	“Due to the complexity of her conditions, the patient was *frequently rejected for treatments or discharged during admissions.* This made the patient feel rejected and desperate because she could never get appropriate help.” “The independent psychiatrist noted that although the patient was very young, her problems were also very complex. The various treatments that the patient had undergone for years *had been without results*. There were *no more reasonable treatment options* and spontaneous improvement seemed impossible.”		“When the tension became too high for her, she damaged herself. There was a *constant risk of suicide.”* “The patient’s GP did not want to take on the patient’s euthanasia request, *for reasons of his own*.” “The doctor found *the patient’s attention could easily be attracted, maintained, and moved.* She had insight into illness … was able to clearly understand the consequences of her choice for euthanasia.” “The patient was *fully competent* according to the criteria of Grisso & Appelbaum … *she looked forward to the irreversible consequences* of her euthanasia request.”
Dutch RTE, 2018-67 Female, 30-40, borderline personality disorder, PTSD, ADHD, anxiety disorder, suicidality	“Healing was *no longer possible*. The treatment was exclusively palliative in nature.” “She *suffered from the hopelessness* of her situation. The patient realized she was unable to make any changes in her problems.” “The doctor was convinced that the patient’s suffering was unbearable and, *according to prevailing medical opinion, without prospect of improvement*.”		“The patient often felt unseen, rejected, and insecure … she had emotional regulation problems with mood swings and *emotional outbursts.*” “Partly because of the patient’s *limited introspective capacity*, there were no further treatment options available for [her eating disorder].” “The patient had previously discussed euthanasia with her GP and treating psychiatrist … *both could not honor* her request … The treating psychiatrist had *fundamental objections*.”
Dutch RTE, 2017-08 Female, 18-30, chronic anorexia (purging subtype), OCD, osteoporosis, depression	“The patient felt trapped between her eating rituals and *untreatable gloominess.”* “The patient had been *exhaustively treated* both for her depressive complaints and for her eating disorder … despite the young age of the patient, *no realistic treatment options* were available.” *“*The patient *actively participated* in all the offered treatments. The treatments had a temporary positive effect on the eating disorders and the depression. However, both recurred quickly after treatment…” *“Healing was no longer possible*. The treatment was solely palliative in nature.” “The physician was convinced that the suffering for the patient was unbearable and, *according to prevailing medical insight, without prospect of improvement.”*		“The *physical deterioration played a role* - the patient was *emaciated*, tired, and dizzy.” “The patient’s GP did not want to perform the euthanasia because *he was not convinced* of the hopelessness of the suffering.” “The patient was *depressed* but not psychotic and (*partly due to her intelligence*) was able to sufficiently reflect on her depressive mood disorder.” “The wish for death was durable and consistent, despite the fact that the patient *had always embraced* the offered therapeutic aids and w*as still willing to try* possible experimental treatments at the time of the conversation.”
Dutch RTE, 2016-76 Female, 60-70, anorexia, osteoporosis, severely underweight	“Unbearable suffering mainly due to physical deterioration, as a result of *long-standing* anorexia.” “The patient was *psychiatrically without any more treatment options* ^7^ *— her* anorexia nervosa was *chronic*.” “*Healing was no longer possible.* The treatment was solely palliative in nature.” “The physician was convinced that the suffering was unbearable, and *according to prevailing medical insight, hopeless.*”		“The patient was *tired and exhausted from her long and ceaseless battle* with anorexia nervosa and did not want to continue living.” “The psychiatrist noted that the psychiatric state of the patient *had been stable* for some years.” “There were *no indications of severe depressive or anxiety disorders*, no psychotic or delirious symptoms, and *no acute suicidal thoughts*.” “There were *no indications of complaints or symptoms that could have influenced her decision-making capacity …* there was no reason to doubt the patient’s decision-making capacity.”
Dutch RTE, 2016-01 Female, 60-70, anorexia, depression, personality disorder, suicidality	“Eventually, no treatments remained that offered a prospect for improvement…. Healing was no longer possible.” “[An independent psychiatrist] concluded that in theory, treatments aimed at the patient’s personality problems *were still possible.* However, the psychiatrist noted that *it was very questionable* whether the patient could handle these treatments and whether she could establish and maintain an adequate treatment relationship.”		“According to the patient, *her spirit had already died earlier.* She was incapable of doing anything.” “The patient felt somber and depressed and was *preoccupied with thoughts of suicide.”* “The patient was *decisionally capable* regarding her request and *considering her limited resilience and poor motivation*, no relevant treatment options remained.”
Dutch RTE, 2015-17 Female, 60-70, anorexia, PTSD, depression, psychosis, suicidality	“She was diagnosed with *chronic* post-traumatic stress disorder (PTSD), *chronic anorexia nervosa*, and *recurrent* depressive and psychotic episodes with self-harm and suicide attempts.” “The patient’s suffering consisted of an *untreatable eating disorder …* the patient could not live with the *untreatable* consequences of her trauma.” “Healing was no longer possible. The treatment was solely palliative.”		“When it became clear that *the general practitioner and a treating psychiatrist would not perform the euthanasia*, the patient registered herself at the SLK^8^.” “She had five discussions with a (first) doctor from the SLK. When it became clear after these discussions that due to a disruption in the relationship between the doctor and the patient, *this doctor could not perform the euthanasia*, another doctor took over the treatment. “The patient’s suffering consisted of the untreatable eating disorder, the *constant high level of anxiety and tension, the (almost daily) severe nightmares with flashbacks …* the psychiatrist found the patient’s euthanasia wish to be comprehensible and respectable. *Her mood was stable.”*
Dutch RTE, 2015-64 Female, 20-30, anorexia, PTSD, depression, self-harm, dissociation, sexual trauma, suicidality	“The patient suffered from *treatment resistant* PTSD and severe *treatment resistant* anorexia nervosa.” “Healing was no longer possible. The treatment was solely palliative.” “The doctor was convinced that the suffering was unbearable for the patient and, *according to prevailing medical insight, without prospect of improvement.* “The patient received various therapies, both outpatient and inpatient, in several expert centers. She was also treated intensively with medications … the patient *had undergone all reasonable treatments* and was considered *to have no more treatment options left* within the current state of medical science.”		“There was also *chronic depression, chronic suicidality, self-mutilation, dissociation/pseudo-hallucinations*, and obsessive thoughts and compulsions. Her complaints started 15 years ago after sexual abuse.” The patient’s suffering consisted of continuous psychological suffering due to *ongoing mood swings and flashbacks*, constant abdominal pain, and a very poor physical condition and deplorable state she had come to be in. “The patient’s wish to die was understandable and realistic, given her prognosis and personality. *There was no depression in the narrow sense or any other mood disorder* influencing her thinking.”
Dutch RTE, 2015-89 Female, 60-70, anorexia, mood issues, alcohol dependency, personality disorder	“The psychiatrist, given the lengthy and intensive treatments the patient had undergone, saw n*o further avenue* for treating her personality issues.” “The psychiatrist noted additionally that *research had shown that persistent feelings of emptiness and detachment are chronic.”*		“The patient *viewed her life as meaningless* and realized that, despite her continuous struggle, there was no prospect of improving her situation.” “She had *attempted to numb her emotions with alcohol and disordered eating*.”
Dutch RTE, 2014-81 Female, 30-40, anorexia, borderline personality disorder, PTSD	“The *failure of treatments* caused tensions for the patient. She then felt the need to punish herself. There were several instances of self-mutilation and self-poisoning.” “Despite the patient’s relatively young age, there was already a lengthy treatment history with a notable downward spiral over the recent years. *Treatment was essentially impossible.”*		“The patient’s suffering consisted of *psychological distress* such as anxiety, panic, and flashbacks of past traumas. Her *physical condition also continued to deteriorate* due to anorexia.”
Dutch RTE, 2013-16 Female, 30-40, eating disorder, borderline personality disorder, kleptomania, OCD, hoarding	“The patient had tried all imaginable psychotherapeutic and pharmacological treatments. Healing was not possible. Treatment options were exhausted.” “The consultant confirmed that the extensive psychiatric issues had proven to be treatment-resistant and thus the suffering was without prospect of improvement.”		“She was a *highly educated woman* who was fully aware of her disorders.” “The patient’s suffering consisted of compulsive thoughts and actions such as eating and purging rituals, the need to move, stealing, and collecting food. Additionally, she suffered from total exhaustion, feelings of anxiety, and depression. She also suffered from *physical deterioration due to starvation.”*
Dutch RTE, 2013-02 Female, 50-60, anorexia, BPD, dissociation	“The patient had *undergone extensive treatment* over the past thirty years, but without the desired result. Eventually, there were *no treatment options left* that offered prospects for improvement.”		“It was *her own explicit wish* and will. She would finally be free from fear and stress.” “[The first consultant] concluded that he did not feel sufficiently capable to determine the pressure of suffering in light of her personality disorder and advised the requesting doctor to seek another opinion.”
Patient B Female, 30-40, eating disorder and personality disorder not specified	*“*[The patient had] treatment-resistant recurrent depression, an eating disorder and a personality disorder not otherwise specified.” “She deeply wanted to grant other people a new chance for a healthy and happy life, *although it was no longer possible* for her.”		“Her brother said that she first spoke to him about her wish to donate her organs after euthanasia, *fearing that this would burden her mother.* He comforted her that dying in the intensive care unit *would not be burdensome for him*.”

5 The Dutch word ‘uitbehandeld’ does not exist in English. It means that there are no more treatment options left.

7 The Dutch Life End Clinic ("Stichting Levenseinde Kliniek") is a mobile clinic that provides euthanasia to patients whose own physicians had declined to perform euthanasia in their cases.

**Table 9:** Other notable features in rationales used in assisted dying for EDs.

	No.	Percent
Clinician rejected or dismissed available treatment options.	9	47%
Condition was deemed irremediable “according to prevailing medical opinion.”	11	58%
Clinician stated treatment would be an undue burden on patient.	5	26%
Patient had been rejected or discharged from prior treatment.	2	11%
Patient was receiving palliative or harm reductive treatment.	9	47%
Patient was underweight/malnourished when request granted.	13	68%
Previous physicians rejected or refused the patient’s request.	8	42%
Patient’s intelligence was cited as evidence of mental capacity	5	26%
Patient changed mind or pushed back dates multiple times.	2	11%
Physician suggested patient sign a DNR order.	2	11%

### 4.5 Irremediability domain

In this domain, ten reasons for assisted dying emerged: (1) the person’s suffering is unbearable, (2) the person’s condition is hopeless, (3) the person has a severe and chronic form of the illness, (4) the person has been ill for a long time, (5) the person’s prognosis is poor, (6) treatment has produced no lasting results, (7) there are no realistic treatment options (8) the person has a treatment-resistant condition, (9) healing is no longer possible, and (10) further treatment would be futile.

The length of time the person had been ill was used as a rationale for assisted death in 95% of all ED cases. Clinicians emphasized the person’s prognosis was poor in 89% of cases, and in 58% of cases, the person’s condition was deemed irremediable “according to prevailing medical opinion.” The failure of past treatments to produce lasting change was used to justify assisted death in 89% of cases. Nearly half, or 47%, of cases stated that healing/cure was not possible for the patient.

In 47% of the cases, terms like “chronic ED” or “severe and enduring anorexia” were cited to justify assisted dying. Patients’ conditions were described as “treatment-resistant” in 42% of cases. In 47% of cases, clinicians either rejected or dismissed existing treatment options, in some cases describing them as an “undue burden” on the patient (26% of cases). Additionally, in 11% of cases patients had been rejected or prematurely discharged from previous treatment attempts. Treatment futility was only cited in the two United States cases (2).

### 4.6 Terminality domain

Three main rationales emerged in this domain: (1) the person has a terminal condition, (2) the person’s death is imminent, and (3) the person is at a terminal stage of illness. Notably, these rationales were used exclusively in the case reports in the United States (where terminality is a legal criterion for MAiD). In both cases in the United States, the patient’s ED was described as a terminal condition, and death was deemed imminent. These cases also suggested the patients were at a terminal stage of anorexia. There were no mentions of terminality in the Dutch case reports.

### 4.7 Voluntary request domain

Six main rationales emerged in this domain: (1) the person has decision-making capacity, (2) the person has been adequately informed of their choices, (3) the decision to die is well-considered, (4) the person has made a consistent request to die, (5) the person’s choices or autonomy should be respected, and (6) the person’s request to die is not a symptom of their mental illness.

The consistency of the request was emphasized in 100% of cases. In two cases (11%), the patient had previously changed their mind or pushed back the date of their assisted death. In all but one case, the patients’ request was described as well-considered, and 84% of cases emphasized the patient was well-informed about their choices.

In 95% of the cases, it was asserted the person had decision-making capacity to end their life through assisted death. Notably, 68% of cases reported the individual as severely underweight or malnourished at the time of their request. Previous physicians had declined the patient’s request for assisted death in 42% of cases. In 26% of cases, the patient’s intelligence was explicitly mentioned as evidence of mental capacity. Similarly, over a quarter of the clinicians (26%) stressed that the patient’s request to die was not a symptom of mental illness. Respect for the individual’s autonomy and choice to die was highlighted in 53% of the cases.

## 5 Discussion

This systematic review reveals critical gaps in both official government reports and peer-reviewed literature on the prevalence of assisted death among patients with EDs. Notably, our findings identified at least sixty deceased patients with EDs who underwent assisted dying, including in countries that restrict the practice to terminal conditions. There is preliminary evidence to suggest that ED patients may be overrepresented among those receiving assisted death for reasons related to mental disorders. Of the 198 Dutch cases where it was possible to discern a psychiatric diagnosis, 16 (8.7%) were patients with eating disorders. In the United States, where terminality is a legal criterion for MAiD, at least four cases of assisted death in persons with EDs were identified (2, 44), raising questions about the integrity of safeguarding procedures. The omission of psychiatric diagnoses from public health data presents major obstacles to understanding this phenomenon, raising significant concerns about oversight and public safety.

Notably, among the cases reviewed for this paper, 100% of patients who underwent assisted death for an ED were female. Studies typically show 69-77% of people who die by psychiatric euthanasia are women (10–12, 96). Although more research is needed to detail gender among the larger population of those with EDs requesting assisted death, these findings suggest the possibility that women may be overrepresented. Accordingly, it is important to investigate the potential role gender bias plays in euthanasia evaluations. Some authors have noted that the nebulousness of criteria like “unbearable suffering” and “irremediable condition” may introduce physician bias and error (10). Nicolini et al. (10) note that Dutch due care criteria encourage clinicians to use their own subjective responses to evaluate the palpability (‘invoelbaar’) of patients’ suffering, and psychiatrists increasingly accept the patients’ subjective definition of irremediable. No jurisdiction mandates the use of standardized mental capacity assessments or psychiatric evaluations before patients undergo assisted dying, which could help protect patients who may be impacted by mental illness (97).

In the cases reviewed, descriptions of patients and circumstances that led to their deaths reflected the legal criteria for assisted dying in the specific jurisdiction. For example, cases in the United States suggested the patients had *terminal* anorexia (2); whereas mentions of terminality were notably absent from Dutch cases, which emphasized the irremediability of patients’ conditions. Critically, while the reports may have been written to align with the legal framework in each jurisdiction, the clinical rationales identified in each case refer to the *medical reasons* each patient’s assisted dying request was granted. In medicine, clinical reasoning requires the conscientious and judicious use of current best evidence to determine a diagnosis, prognosis, and decisions about the care of individual patients (100, 101). The following section discusses the empirical literature on each clinical rationale identified in the review.

### 5.1 Irremediability domain

In 95% of cases, patients who underwent assisted dying were described as having irremediable, incurable, or untreatable EDs. More than half (58%) of cases stated the patients’ conditions were irremediable with a high degree of medical certainty. For example, “The physician was convinced that the suffering for the patient was unbearable, and, *according to prevailing medical insight*, without prospect of improvement” (2017–08). Nearly half (47%) of the reports included the phrase, “Healing was no longer possible.” In many cases, unbearable suffering was emphasized as a way of conveying the irremediability of the condition.

Prognostic accuracy is a significant challenge for psychiatry, and research by Ferrand et al. (23) indicates that physicians’ predictions of prognosis are often inaccurate, with a trend toward pessimism. Medical professionals’ perception of people with psychiatric disorders may also negatively impact prognostic predictions and reduce endorsement of recovery-oriented treatments (102). Clinical rationales in this domain utilized underdeveloped concepts which lack empirical validity in the ED literature, including clinical stages of EDs, treatment futility, treatment resistance, and illness duration as a predictor of poor prognosis. Across this domain, there is evidence in the empirical literature that directly contradicts the clinical rationales physicians used to justify assisted dying in patients with EDs.

#### 5.1.1 Severe and chronic form of illness

Nearly half of cases (47%) suggested patients who underwent assisted death had a severe and chronic form of illness. Specifically, patients were characterized as having severe and enduring eating disorder (SEED), chronic anorexia, severe and enduring anorexia (SE-AN), and *terminal anorexia.* A poignant example is provided by Gaudiani et al. (2), in a passage where a patient, now deceased, recounts her experience:

“As a patient with *severe and enduring anorexia nervosa* advocating for my legal right to MAID, I confronted numerous obstacles and challenges from the medical profession, related not just to the question of whether I should have access to MAID generally, but more so, how my anorexia — a psychiatric condition frequently misunderstood by the medical community — interacted with my decision making capacity and desire to pursue MAiD as one potential option *knowing that my illness was indeed terminal*” (p. 1).

Emerging from literature seeking to delineate and develop targeted treatment approaches for patients at different stages of illness, terms like SEED and SE-AN represent an effort to develop clinical staging models for EDs, in which progressive stages of the disorder present unique characteristics (5, 103, 104). Staging models are thought to be a refined approach to diagnosis linked to a continuum of symptomatology, which may help identify effective interventions and treatment needs (105–107). Despite a growing and laudable body of work in this area, attempts to empirically validate distinct clinical stages based on severity, chronicity, and response to treatment have been unsuccessful (108). Further, there is little consensus on the operational definitions of these terms; criteria for what constitutes severity, chronicity, and lack of response to treatment for the nosological labels SEED and SE-AN vary widely between studies. They have not been validated for clinical use or formally recognized as a psychiatric diagnosis by the International Classification of Diseases, 11^th^ Edition (ICD-11) or Diagnostic and Statistical Manual of Mental Disorders, 5^th^ Edition, Text Revision (DSM-5-TR) (105, 108–112).

In this study, terms like SEED and SE-AN were used in a *pseudo-diagnostic* capacity; that is, they were discussed with patients and families as if they were legitimate clinical categories and used to guide treatment decisions. For example, one patient was described as: “*diagnosed with* chronic PTSD, chronic anorexia nervosa, and recurrent depressive and psychotic episodes with self-harm and suicide attempts” (2015-17, emphasis added). In some cases, this may have led to potentially misguided treatment decisions, such as prematurely discontinuing curative forms of treatment (e.g., weight restoration and inpatient hospitalization) and dismissing treatment options with reasonable chance of success (e.g., involuntary hospitalization or placement of a feeding tube) based on the perceived “stage” of illness (2 p. 8).

The cases in this review illustrate how patients may come to view their illness as irremediable through a gradual process influenced by clinical, familial, and societal interactions. This may progress from an initial diagnosis of an ED to identification with a term like SEED or SE-AN, culminating in the perception of their condition as irremediable, and in some cases terminal (2). Assisted dying decisions are shaped by the opinions, attitudes, and beliefs of others, particularly those in medical authority. The use of these terms *as if* they were formal diagnoses may have introduced subtle coercive effects and impaired patients’ ability to make voluntary decisions. According to the Netherlands due care criteria, “a patient cannot make a well-considered decision without a full understanding of the disease, diagnoses, prognoses, and treatment options. It is the physician’s responsibility to ensure that the patient is fully informed” (113). As illustrated by case examples, misunderstanding and misidentification may alter how patients understand their condition and treatment choices. Critically, *understanding* is a fundamental component of decisional capacity, and in most countries, patients must *fully understand* their diagnosis, prognosis, and treatment choices to *voluntarily consent* to assisted death.

#### 5.1.2 Illness duration and poor prognosis

In many cases, the length of time the person had been ill was juxtaposed with past treatment failures to suggest that further attempts at treatment were unlikely to be successful. For example, a Dutch case report suggested: “Despite the patient’s relatively young age, there was already a lengthy treatment history with a notable downward spiral over the recent years. *Treatment was essentially impossible”* (emphasis added, 2014-81).

Research on the relationship between illness duration and treatment outcomes EDs has yielded inconclusive results. Current evidence largely indicates that the length of time a person has been ill is not a reliable predictor of recovery outcomes (114). Although some studies have identified a correlation (115–117), a recent meta-analysis found no significant relationship between illness length and response to treatment (114). To illustrate, Calugi et al. (118) found that patients with an illness duration of seven or more years showed comparable rates of recovery to those with shorter durations of illness at 12-month follow-up. Steinhausen (116) found that while duration of illness was a predictor of poor outcomes, studies that used a longer duration of follow-up showed better outcomes. Longitudinal studies suggest that most people with EDs experience a protracted course of illness and recovery, and many patients endure multiple treatment failures before achieving remission (119). The few studies that have followed patients for at least 20 years suggest patients continue to recover a decade or more after illness onset. For instance, Eddy et al. (119) followed 121 patients with anorexia and assessed rates of remission 9 years and 22 years after initial presentation, finding that approximately a third had recovered by the 9-year mark, and by the 22-year follow-up, two-thirds had fully recovered. Critically, they found that 50.6% of those who had not recovered nine years in had attained recovery by 22-year follow up.

#### 5.1.3 Treatment resistance

The construct of *treatment resistance* was referenced in 42% of cases to suggest that patients had an irremediable condition, unlikely to improve with treatment. For example, a report describing a patient in her twenties with co-occurring anorexia nervosa, major depressive disorder, and avoidant personality disorder stated: “the patient’s *inability and unwillingness to live* was assessed by the doctor as persistent and treatment resistant” (2023–67). Peer-reviewed case series on assisted dying in EDs cited similar justifications. In a study reporting on ten patients with EDs who were euthanized in Belgium, the authors stated: “in all patients, the suffering was *chronic, constant and unbearable*, without prospect of improvement, due to *treatment resistance*” (12 p. 5).

Terms such as “treatment resistant” (120 p. 247), “treatment refractory” (121 p. 372), “recalcitrant” (122), and “severe and intractable” (123) have appeared frequently in ED research and commentaries, often within sweeping statements suggesting patients who have not recovered within a number of years are unlikely to improve with further treatment. Authors rarely make a distinction between *a patient who has not yet recovered* and *a patient who is unable to recover*. Conceptions of treatment resistance vary widely between studies (124). Studies have defined treatment resistance as an illness persisting greater than 7-10 years (124), patterns of multiple readmissions to ED services (125), one unsuccessful course of therapy (126), immediate weight relapse after treatment (127), or a pattern of increasing medical instability lasting at least two years following expert treatment that included at least two involuntary feedings (127). Inaccurately, many authors use the term treatment resistance interchangeably with chronicity of illness or difficulty to treat, muddying the clinical validity of the term and complicating efforts to generalize findings between studies (128).

Critically, conceptions of treatment resistance also do not distinguish between patients who elect to end treatment for reasons pertaining to the treatment itself and those who ended treatment early due to financial constraints (e.g., inadequate insurance), work and family responsibilities (e.g., lack of childcare or risk of job loss), or because the treatment provider prematurely ended care (e.g., inability to manage complex medical needs) (125, 129). At least two of the patients who underwent assisted dying identified in this review had been previously rejected from treatment or discharged early (2022–85) (2). This raises the question of whether treatment resistance was emerging from providers rather than the patients themselves. In one case, a particularly young patient (aged 18-30) described as having “chronic anorexia” and “untreatable gloominess” expressed a willingness to attempt further treatments during her assessment for euthanasia (2017–08). The report stated: “The patient had always embraced the offered therapeutic aids and was still willing to try possible experimental treatment forms at the time of the conversation.” Despite having shown improvements from previous treatments, it concluded: “Healing was no longer possible. The treatment was solely palliative in nature.”

Some authors have suggested that the problem of treatment resistance lies not in patients with untreatable conditions, but rather in healthcare systems unable to provide effective treatment. In an article synthesizing the perspectives of clinicians, carers, and people with EDs, Downs et al. (87) suggest that resistance to treatment is not a characteristic of the patient or their condition, but rather a product of failures in the healthcare system more broadly to treat the person and their condition effectively. In countries like the Netherlands and United Kingdom where publicly funded ED services are often overstretched; some patients report being denied treatment after being labeled treatment resistant. Downs et al. (87) writes:

​​“I have spent over a decade of my life either waiting for or being denied ED treatment when asking for it … I endured all of this time without care only to be told when it was available that I was resistant to treatment, and even, that I would live with an ED for the rest of my life at best and die at worst” (p. 150).

The term *treatment resistant* assumes that research has established what constitutes empirically supported treatment for EDs and that treatments are regularly implemented with fidelity across care settings. Unfortunately, few treatments have been empirically studied in patients with severe and chronic EDs. To illustrate, Zhu et al. (130) reviewed psychological treatments for individuals with SE-AN and found only two demonstrated limited evidence of efficacy (i.e., special supportive clinical management and cognitive behavioral therapy for anorexia). Studies also show treatment fidelity varies widely between clinicians and approaches often do not align with evidence-based practice, even when the therapy is described as such (131, 132).

#### 5.1.4 Lack of treatment options

In 17 out of 19 documented cases, reports emphasized that no realistic treatment options remained for the patients who underwent assisted death. Notably, 16 of the patients resided in the Netherlands, where legislation mandates that physicians confirm with the patient there are no reasonable treatment alternatives to euthanasia. Although patients are not required to exhaust every conceivable treatment, those who decline a reasonable alternative are ineligible for euthanasia (9). However, some studies indicate that these guidelines allow for significant physician discretion (9, 10). For instance, Nicolini and colleagues (10) reported that 28% of euthanized patients with personality disorders had not undergone psychotherapy, and 27% had never been hospitalized, yet the Regional Euthanasia Review Committees (RTE) deemed that the physicians had complied with due care guidelines.

In line with findings by Nicolini et al. (9), we found that in 47% of cases, the consulting physicians acknowledged there were possible treatments which had not been tried but dismissed them as unlikely to succeed. For example, a psychiatrist in one Dutch case posited that while treatments targeting the patient’s personality issues were theoretically possible, “it was very questionable whether the patient could handle these treatments and whether she could establish and maintain an adequate treatment relationship” (2016-01). In a case involving a patient with anorexia who had never weight restored nor completed residential treatment, the patient’s parents asked the consulting physician if other treatment options (e.g., a feeding tube) should be tried before MAiD. The physician responded, “If someone restricts the tube God gave them, (i.e., their esophagus), they would also be very likely to restrict [their food] through a surgical feeding tube, so that would not be a long-term solution” (2 p. 9).

Our findings are consistent with previous reports showing most psychiatric patients requesting euthanasia have multiple diagnoses (8, 10, 12). Sixty-one percent had more than three comorbid psychiatric disorders, and 22% had four or more psychiatric conditions. However, in some cases, comorbid conditions were not treated before proceeding with euthanasia. For instance, in one case, a consulting physician acknowledged that the patient’s autism had not been addressed in prior treatment plans and then stated the patient would not benefit from psychotherapy due to her “lack of reflective and mentalizing capacity” (2023-34). In another case involving a person with autism, the psychiatrist stated: “Treatment aimed at autism spectrum disorder was not used, because that was not the biggest problem and because ASD cannot be treated in a therapeutic sense” (2023-04). This contradicts evidence suggesting that adapting treatment strategies to accommodate neurodivergent individuals (e.g., accounting for sensory processing differences and communication challenges) can enhance treatment outcomes (88, 133, 134). For example, the Pathway for EDs and Autism developed from Clinical Experience (PEACE) pathway, designed for patients with concurrent diagnoses of anorexia and autism, has demonstrated early cost-savings and favorable clinical results (133).

In 26% of cases, clinicians dismissed available treatment options as posing an “undue burden” on the patient. One report, for example, described a patient in her twenties who had been ill for nine years and not achieved “lasting results’’ through psychotherapy and medications (2023-67). The patient’s GP had declined her request for euthanasia, but a consulting psychiatrist at a euthanasia center granted it, stating: “The chance of recovery was estimated as small and it was disproportionate to *the great burden on the patient*” (emphasis added, 2023-67).

#### 5.1.5 Treatment futility

The notion that further treatment is futile was used as a rationale for assisted death in both case studies from the United States, but it was notably absent in cases from the Netherlands. This may be because the Dutch due care criteria only require physicians to confirm with patients there is “no reasonable alternative” to euthanasia (81). In the case studies in the United States, futility figured prominently in the rationale for prescribing MAiD. For instance, Gaudiani et al. (2) suggested that two women with anorexia who were prescribed MAiD were eligible in part because they “understand further treatment to be futile” (p. 11).

Futility is a contentious concept in medicine, and only a small number of commentaries have explored its application to psychiatric conditions (135). Physicians use futility judgments to determine whether to pursue treatments that offer minimal benefit and pose significant risks to the patient (136). While the term is colloquially used to mean pointless or useless, in medicine it is defined operationally in three ways. A treatment that is deemed *physiologically futile* cannot physically achieve the desired effect in the patient; one that is *quantitatively futile* has 0-2% chance of working; and a *qualitatively futile* treatment is likely to produce such poor outcomes it is deemed best not to attempt it (137–139).

Importantly, futility refers to the likelihood of a specific treatment to benefit a patient at a particular time; it does not apply broadly to whether a patient can benefit from any treatment (140). This nuance was lost in the cases described by Gaudiani et al. (2), which emphasized that to be eligible for MAiD, patients must “understand further treatment to be futile” (p. 11). Notably, the responsibility for assessing treatment futility was predominantly placed in the hands of the patients themselves, rather than the medical professionals responsible for their care (2). Both patients described in the study were severely underweight and depressed, and one reported active suicidal ideation (2).

Many bioethicists have questioned the empirical and ethical basis for applying futility in psychiatry (141–143). For example, Geppert (141) highlighted that even the worst outcomes in ED treatment do not meet the quantitative futility threshold used in medicine (<2% chance of success). Other authors noted that prognostic accuracy is so poor in psychiatry that futility judgments depend almost entirely on subjective opinion and may be influenced by the patient’s access to and ability to pay for treatment (141, 142).

### 5.2 Terminality domain

In cases in the United States, where a terminal prognosis is a legal prerequisite for assisted death, authors asserted that ED patients prescribed MAiD had terminal conditions (2, 5). Specifically, Gaudiani et al. (2) proposed these patients represent a clinical subcategory of SE-AN: terminal anorexia. They posited that for these patients, death was inevitable (2). The authors argued that delineating a new terminal stage of ED could enhance access to palliative care and MAiD in states where it is legally permitted. The proposed clinical characteristics of terminal anorexia included: “A consistent, clear expression by an individual, who possesses decision-making capacity, that they understand further treatment to be futile, they choose to stop trying to prolong their lives, and they accept that death will be the natural outcome” (2, p. 11).

Notably, this conception of terminality deviates from medical definitions of a terminal condition, by describing reversible cognitive behaviors (thoughts, thinking patterns, cognitive distortions) as indicators of a terminal illness (i.e., an *understanding* that treatment is futile, *choosing* to stop trying, and *accepting* death). Medical texts describe the terminal phase of an illness as a period of *inexorable* and *irreversible* decline leading to death, with *no expectation of recovery* and a survival prognosis of only months or less (144, 145). Unlike cancer or Alzheimer’s disease, most medical complications associated with anorexia can be treated with adequate nutritional intake and weight restoration, even in severely emaciated patients (3, 146). Furthermore, cognitive and emotional symptoms associated with anorexia, such as despair and cognitive distortions, also improve with effective treatment (147–149).

Notably, California and Colorado’s annual reports showed no record of the two aforementioned deaths described by Gaudiani et al. (2). The manager of the Colorado’s Vital Statistics Program confirmed that anorexia has been reported as a terminal condition in previous years and reported in the undefined category of *Other Illnesses* (98). Crucially, he stated that CDPHE lacks authority to investigate potentially suspicious reports if there are concerns a prescriber may be misrepresenting a psychiatric illness as a terminal condition (98). This raises significant concerns about the inadequacies of current safeguards to ensure public safety, especially for vulnerable groups. Publicly available reports obscure many of the details needed to monitor trends and identify potential violations (73). Reports do not currently list co-occurring psychiatric diagnoses, prescriber(s) of MAiD, health insurance status, and patient characteristics associated with each condition (73).

### 5.3 Voluntary request domain

In all cases reviewed, reports indicated that patients had voluntarily chosen to die. For a decision to be voluntary, an individual must have adequate decision-making capacity (understanding), make a deliberate decision (intentionality) (3) be expressing their genuine desires (authenticity), and make the decision free from controlling or coercive influences (independence) (**Figure 2**). While clinical rationales asserted that patients were capable of making a life-ending decision and that their desire to die was not influenced by symptoms of mental illness, literature suggests that capacity impairments can be subtle in EDs (42, 150). There is evidence that clinicians might not always accurately assess these impairments (42, 150). Furthermore, ED-related psychopathology may compromise patients’ ability to discern and communicate their true wishes, raising questions about the authenticity of the voluntariness of their decision to die.

#### 5.3.1 Decision-making capacity

The capacity to make a voluntary request for assisted death hinges on the patient’s ability to understand, appreciate, reason, and communicate information specific to the decision to end their life with medical assistance (52). Although clinicians reported adequate decision-making capacity in 95% of cases, the specific methods used to assess this (e.g., standardized tests, clinical assessments) were not detailed. Moreover, 42% of these cases had previously been rejected by other physicians, although it was not always clear why they had rejected the patient’s request.

Notably, records from 68% of cases indicated that patients were malnourished at the time of their request. This raises profound concerns, given that research has documented extensive cognitive impairments associated with malnutrition in anorexia nervosa, including brain atrophy, cognitive distortions, reduced executive functioning, and emotional processing issues (151–155). These impairments contribute to a vicious cycle where malnutrition exacerbates cognitive deficits that hinder effective treatment engagement and decision-making, resulting in a pattern of diminished capacity that fuels the disorder itself (146).

Despite extensive research on decision-making capacity in patients refusing treatment, there is a notable lack of studies specifically addressing capacity in the context of assisted dying decisions. There is preliminary evidence from the research on capacity to consent to treatment to suggest this line of inquiry is worth pursuing further. Previous research indicates that decision-making impairments in patients with EDs are often most pronounced in their ability to appreciate the consequences of their decisions (42). Diminished capacity correlates strongly with alexithymia, suggesting emotional deficits may be a mediating factor (42).

The challenge of accurately determining mental capacity is compounded by the clinical reality that patients with EDs may appear lucid yet lack insight into their disorder and its life-threatening risks (149). In 26% of cases, clinicians highlighted patient intelligence as evidence of capacity, which may reflect bias or misunderstanding in clinical assessments. Past studies have found significant variability and potential bias in clinical assessments of mental capacity in patients with EDs (42, 156). For instance, discrepancies between clinicians and standardized tests have been observed, with little consensus on which patients lack capacity (150). Notably, there are no requirements for standardized mental capacity assessments in assisted dying legislation across various jurisdictions.

Overall, the evidence indicates variable and, at times, impaired mental capacity among individuals with EDs, along with a risk of biased clinical judgments, suggesting a critical need for more rigorous and standardized assessment methods in the context of assisted death.

#### 5.3.2 Impact of psychopathology on autonomous expression

**Figure 6:**
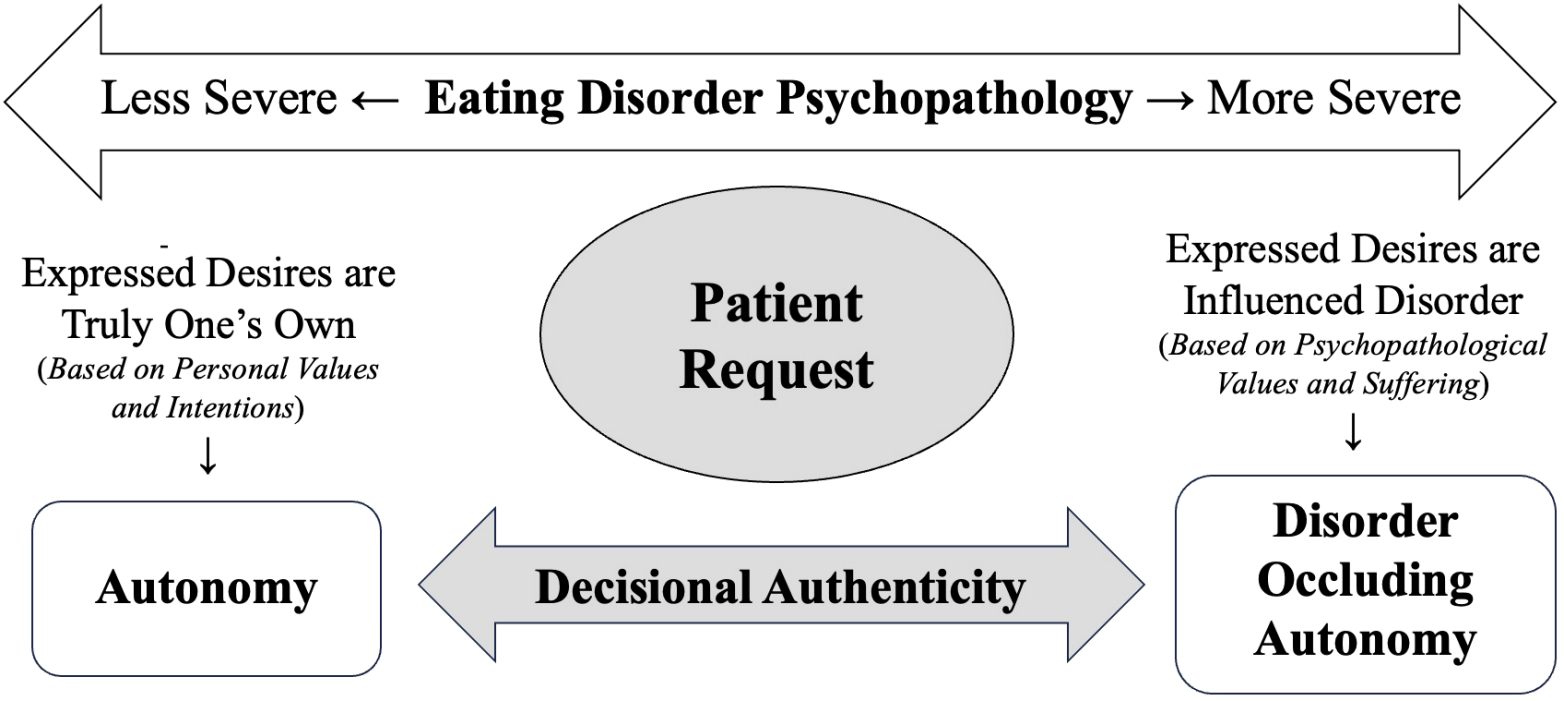
Autonomy, decisional authenticity, and psychopathology. Informed by Cook-Cottone (157) and Sjöstrand and Juth (35).

For a request for assisted death to be considered voluntary, a patient must be capable of determining their true wishes (29). A request is not voluntary if, for example, it arises from symptoms of a psychiatric disorder (29). In our review, 53% of cases emphasized the autonomous nature of the patient’s decision, and in 26% of cases, clinicians explicitly stated that the patient’s decision to die was not a symptom of their mental illness. However, theories and research indicate that EDs can profoundly distort a patient’s self-perception and temporarily impair their ability to authentically express autonomy (**Figure 6**) (157). Sjöstrand and Juth (35) describe this as a lack of *decisional authenticity*, distinct from a lack of capacity. Even if individuals with EDs can reason effectively, their decision-making may be so heavily influenced by their pathology that it fails to reflect their true desires (14, 40, 157–162).

In such cases, the request to die may be a remediable symptom of a psychiatric disorder, rather than a genuine expression of the person’s wishes. In order to assess the voluntariness of a decision, clinicians must carefully differentiate whether verbal or behavioral expressions by the patient reflect underlying psychopathological symptoms (e.g., depressive thinking, anhedonia, alexithymia, and suicidality) or an authentic expression of autonomy (35, 88).

The gradual subjugation (or disappearance) of a person’s concern for their own life during acute experiences of ED have been published across theoretical models of the disorder for almost two decades (157, 158, 163). Tan et al. (161) suggest patients with AN may adopt altered, pathological values during their illness, which typically subside upon recovery. Elliot (159) suggests that if a patient does not have a minimal degree of concern for their own life, then a fundamental assumption underlying informed consent is undermined, as it assumes the patient assesses risks with at least some concern for their welfare. Ascribing autonomy to the expression of symptoms of a treatable mental disorder in order to grant a request for assisted dying reflects a fundamental misunderstanding of the nature of EDs and their associated psychopathology and an obfuscation of a physician’s duty to prevent harm (non-maleficence).

Psychological despair in patients may manifest as behaviors that seem autonomous and self-determined, like a wish to die or to lose weight even if it means risking death. Such expressions, however, arise from a chronically malnourished brain and significantly diminished quality of life, accompanied by a lack of awareness of the ongoing impact of starvation. High rates of depression and suicidality among individuals with AN further complicate the interpretation of autonomy (160, 162). Consistently, our review found substantial rates of major depression (47%), depressive symptoms (89%), suicidal ideation (58%), and past suicide attempts (37%), all of which can overshadow genuine autonomy with psychopathological influences (35, 88).

## 6 Conclusions and future directions

The results of this systematic review underscore considerable gaps in the reporting of assisted death in patients with psychiatric conditions, posing substantial concerns about oversight, patient safety, and the ability of researchers to assess the effectiveness of safeguards across different jurisdictions. The clinical rationales used to justify assisted death in patients with EDs were examined in three domains — irremediability, terminality, and voluntary request — and critically assessed against the backdrop of existing research on eating disorders. These rationales used concepts that lack rigorous standardization and validity (e.g., SEED and SE-AN, treatment resistance, and clinical staging) and sometimes directly contradicted empirical evidence (e.g., illness duration as a predictor of poor prognosis and futility of treatment), rendering their application in assisted death decisions questionable. To our knowledge, this study was the first to systematically examine the rationales physicians use to grant patients with eating disorders access to assisted dying. Future studies are needed to extend this research to other psychiatric conditions, as well as investigate rationales for refusing patients’ requests for assisted dying.

To enhance research on assisted dying for individuals with EDs, robust and transparent reporting in public health records is essential. This data is crucial to determine the prevalence of assisted death in EDs and evaluate the empirical foundations of the clinical rationales outlined in this review (88). Further, this information will ensure appropriate oversight so that any potential misapplications of eligibility criteria or violations of assisted dying statutes can be identified and vulnerable groups are protected. Future research must be inclusive of individuals with lived experience of both illness and recovery, as well as those with psychiatric comorbidities (e.g., depression, suicidality, trauma, and PTSD) and diverse identities (e.g., neurodivergence and LGBTQ+) (88).

There is a pressing need for a robust, empirically informed framework to guide care for patients with EDs who express a wish to die. Such a framework must navigate the delicate balance between respecting a person’s choice (autonomy) and the ethical obligations to provide help (beneficence) and avoid harm (nonmaleficence) (140). In the context of assisted death, the relationship between ED symptomatology, mental capacity, and autonomy is fraught. Similarly, determining what constitutes harm and benefit in eating disorder care is not straightforward – those who have experienced multiple treatment failures may be reluctant to engage in further treatment and more inclined to request assistance in death. They may feel the process of regaining weight is so physically painful and emotionally distressing that not existing at all is preferable to enduring treatment (163). Paradoxically, in order to help patients with eating disorders recover, clinicians must encourage them to engage in the activity that distresses them the most (i.e. eating).

Given the gaps in empirical support for rationales highlighted in this review, it is critical clinicians and oversight agencies scrutinize and challenge the legal and ethical basis for the use of assisted death in patients with EDs. Without rigorous, standardized validation of the concepts used in these rationales, the risk of misapplying assisted death protocols and the potential for irreversible harm is high. Finally, while more research is needed to develop an empirically informed framework for evaluating patients who request assisted death, this review also highlights a more urgent need the development and expansion of treatment approaches specifically for patients with severe and chronic EDs. While the discourse on assisted death for patients with EDs has catalyzed an important conversation in the field, it risks diverting attention and resources away from what patients with EDs need the most: consistent, responsive, and effective treatment to help them to recover and lead meaningful lives.

## Data availability statement

The original contributions presented in the study are included in the article/**Supplementary Material**. Further inquiries can be directed to the corresponding author.

## Author contributions

CR: Conceptualization, Investigation, Visualization, Writing – original draft, Writing – review & editing, Data curation, Methodology. CC: Investigation, Supervision, Writing – original draft, Writing – review & editing, Conceptualization, Methodology.
