# Supplementary material for: Assisted death in eating disorders: a systematic review of cases and clinical rationales
Source: Front Psychiatry. 2024 Jul 31;15:1431771. doi: 10.3389/fpsyt.2024.1431771 (PMC11322357; doi:10.3389/fpsyt.2024.1431771)
Supplement: Supplementary file 1 [file Table_1.docx]

**Supplemental Table 1. Databases and Search Queries Used in the Scoping Review**

| Search Database | Query |
| --- | --- |
| Pubmed | (("Euthanasia"[MeSH Terms] OR "suicide, assisted"[MeSH Terms] OR "Right to Die"[MeSH Terms] OR euthanasia* OR "assisted suicide*" OR "assisted dying" OR "assisted death*" OR "assistance in dying" OR "aid in dying" OR "physician assistance in dying" OR "physician assisted death*" OR MAID*) AND ("Feeding and Eating Disorders"[MeSH Terms] OR anorexia* OR bulimia* OR "eating disorder*")) NOT ("Animals"[MeSH Terms] NOT ("Animals"[MeSH Terms] AND "Humans"[MeSH Terms])) |
| APA PsycInfo | (((DE "Euthanasia") OR (DE "Assisted Suicide")) OR (TI (euthanasia* OR assisted suicide* OR assisted dying OR assisted death* OR assistance "in" dying OR aid "in" dying OR physician assistance "in" dying OR physician assisted death* OR MAID* OR Right "to" Die) OR AB (euthanasia* OR assisted suicide* OR assisted dying OR assisted death* OR assistance "in" dying OR aid "in" dying OR physician assistance "in" dying OR physician assisted death* OR MAID* OR Right "to" Die))) AND ((DE "Eating Disorders" OR DE "Anorexia Nervosa" OR DE "Avoidant/Restrictive Food Intake Disorder" OR DE "Binge Eating Disorder" OR DE "Bulimia" OR DE "Feeding Disorders" OR DE "Hyperphagia" OR DE "Kleine Levin Syndrome" OR DE "Orthorexia" OR DE "Pica" OR DE "Purging (Eating Disorders)" OR DE "Rumination (Eating)") OR (TI (eating disorder* OR anorexia* OR bulimia*) OR AB (eating disorder* OR anorexia* OR bulimia*))) |
| Web of Science | ((TS=(Euthanasia* OR assisted suicide* OR assisted dying OR assisted death* OR assistance in dying OR aid in dying OR physician assistance in dying OR physician assisted death* OR MAID*) AND TS=(anorexia* OR bulimia* OR "eating disorder*"))) NOT (TS=((animal* OR rat OR rats OR mouse OR mice OR murine OR dog OR dogs OR canine OR cat OR cats OR feline OR rabbit OR cow OR cows OR bovine OR rodent* OR sheep OR ovine OR pig OR swine OR porcine OR veterinar* OR chick* OR zebrafish* OR baboon* OR nonhuman* OR primate* OR cattle* OR goose OR geese OR duck OR macaque* OR avian* OR bird* OR fish*) NOT (human* OR patient* OR women OR woman OR men OR man))) |
| Academic Search Complete | ( (DE "EUTHANASIA" OR DE "KILLING of people with mental illness" OR DE "KILLING of the elderly" OR DE "PASSIVE euthanasia") OR (DE "ASSISTED suicide") OR TI ( euthanasia* OR assisted suicide* OR assisted dying OR assisted death* OR assistance in dying OR aid in dying OR physician assistance in dying OR physician assisted death* OR MAID* OR Right to Die ) OR AB ( euthanasia* OR assisted suicide* OR assisted dying OR assisted death* OR assistance in dying OR aid in dying OR physician assistance in dying OR physician assisted death* OR MAID* OR Right to Die ) ) AND ( DE "EATING disorders" OR DE "ANOREXIA nervosa" OR DE "BINGE-eating disorder" OR DE "BULIMIA" OR DE "COMPULSIVE eating" OR DE "COPROPHAGIA" OR DE "EATING disorders in women" OR DE "HYPERPHAGIA" OR DE "ORTHOREXIA nervosa" OR DE "PICA (Pathology)" OR TI ( eating disorder* OR anorexia* OR bulimia* ) OR AB ( eating disorder* OR anorexia* OR bulimia* ) ) |
